# Automated synthesis of ^18^F radiolabelled indole containing Oncrasin-like molecules; a comparison of iodonium salts and boronic ester chemistry

**DOI:** 10.1186/s41181-020-00104-x

**Published:** 2020-11-09

**Authors:** Alexander F. McDonald, Yit Wooi Goh, Jonathan M. White, Andrew M. Scott, Uwe Ackermann

**Affiliations:** 1 The Olivia Newton-John Cancer Research Insititute, and School of Cancer Medicine, La Trobe University, Heidelberg, 3084 Australia; 2 Department of Molecular Imaging and Therapy, Austin Health, Heidelberg, 3084 Australia; 3 School of Chemistry, Bio21 Institute, The University of Melbourne, Parkville, 3010 Australia; 4 Melbourne, Australia; 5 Faculty of Medicine, The University of Melbourne, Parkville, 3010 Australia

**Keywords:** Radiofluorination, ^18^F fluorination, Automated synthesis, Oncology, Tracer development, Iodonium salt, Boronic ester

## Abstract

**Background:**

Oncrasin-1 is a small molecule which was identified from a screen of KRAS mutant cancer cells and has shown specificity for KRAS mutant cell killing. We aimed to develop a radiolabelled form of Oncrasin-1 to enable in-vivo imaging of mutant KRAS expression in malignant tumours. This work outlines the synthesis of 3 fluorinated derivatives and development of iodonium salt and boronic ester precursors for radiolabelling with the ^18^F isotope.

**Results:**

In our hands, synthesis of iodonium salts were not easily accessible due to the 3-carbaldehyde indole structure being preferentially oxidized by conditions required for iodonium salt formation, rather than benzyl iodide. Synthesis and radiolabelling of boronic acid pinacol ester precursors were successful, with the products being obtained in yields of 10.76% ± 0.96% (*n* = 5), 14.7% ±8.58% (*n* = 3) and 14.92% ±3.9% (n = 3) for ^18^F KAM001, ^18^F KAM002 and ^18^F KAM003 respectively, with radiochemical purity of greater than 99%.

**Conclusions:**

The successful synthesis of these tracers has been undertaken utilizing boronic ester radio-fluorination methods and will allow for investigation of Oncrasin based molecules as potential diagnostics for cancers expressing mutant KRAS protein.

**Supplementary Information:**

**Supplementary information** accompanies this paper at 10.1186/s41181-020-00104-x.

## Background

Human RAS genes have been identified as one of the most frequently mutated oncogene family in all cancers, with estimations of up to a third of all cancers harbouring a mutation (Baker and Der 2013). Normal function of this family includes regulation of transcription, cell cycle progression, growth, survival, proliferation and cell migration signalling (Downward 2003). For the KRAS protein, single point mutations at positions 12, 13, and 62 have been shown to result in constitutively active protein which in turn causes over expression or increased activity in a wide range of downstream signalling pathways (Bos 1989).

The high frequency of mutation in cancer has made this gene family a target of significant interest within the field of oncology, however, despite extensive efforts to exploit these proteins as potential therapeutic targets, molecules designed for RAS mutant therapies have not made progress into the clinic.

**Fig. 1:**
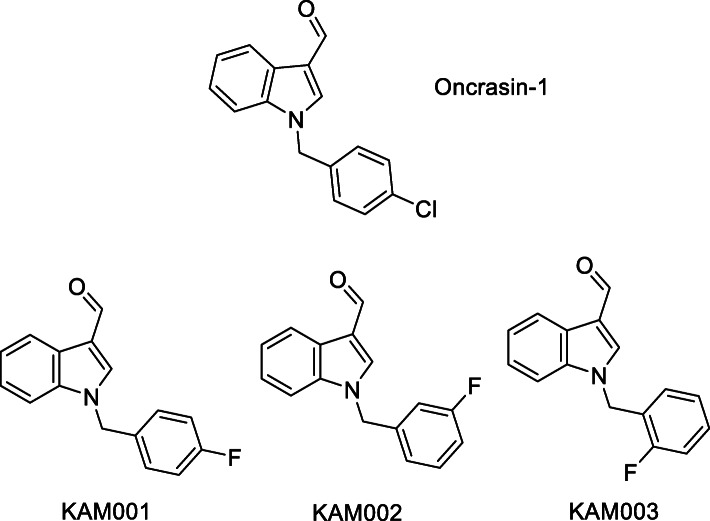
Parental compound, Oncrasin-1, and 3 fluorinated derivatives synthesized

Since identification of this oncogenic driver, efforts to develop new molecules to target KRAS have been ongoing; a molecule, denoted Oncrasin-1 shown in Fig. 1, being discovered in 2008 through a synthetic lethal screen with KRAS mutation in breast cancer cell lines (Guo et al. 2008). In the initial screen the compound showed selective toxicity in both a KRAS mutant cell line and a cell line with wild type KRAS which was previously resistant but became sensitive after transfection with mutant KRAS.

Further optimization of the structure has been reported, with some analogues and derivatives showing improved potency against the KRAS mutant cell lines, including analogues with fluorine at the *meta* and *ortho* positions on the benzyl ring (Wu et al. 2011), shown in Fig. 1.

As these compounds can potentially identify mutant KRAS in different cancer types, they may have potential for development as a diagnostic imaging approach for stratification of patients to different treatment groups. This would be a valuable tool for treatment planning as the presence of KRAS mutant protein is a predictor of poor response to anti-EGFR antibody treatments such as Cetuximab and Gefetenib, due to EGFR-independent downstream signalling through this pathway (Massarelli et al. 2007; Lievre et al. 2006; Karapetis et al. 2008).

Positron emission tomography (PET) is an imaging technique which enables non-invasive imaging of metabolic processes and biochemical function in living tissues. PET imaging currently plays a central role in the detection, staging and response assessment of cancer patients, with clinical management based on based on PET imaging results being an accepted part of cancer patient care.

Radiosynthesis of ^18^F substituted aryl systems is currently a significant challenge in the field of radiofluorination, with limited means to effectively incorporate a radiolabel into an aryl system (Preshlock et al. 2016; Mossine et al. 2015). Many methods which are used for synthesis of ^19^F fluorination of aryl systems rely on electrophilic sources of fluorine, which are not easily accessible when working with ^18^F, or protracted reaction times, which are unacceptable in the context of a 109.7-min half-life. Preliminary evaluation of radiosynthetic methods indicated a limited number of potential precursors for fluorination reactions which position, which cannot be accessed through traditional electrophilic aromatic substitution radiochemistry. All three substitution patterns present a potential radiotracer target, therefore, establishing a method which could be used for radiofluorination reactions at all three positions is essential. To this end, Iodonium salt chemistry has become an area of great interest for radiofluorination of aryl systems, with many publications providing synthetic routes which have allowed access to radiotracers through this chemistry (Preshlock et al. 2016; Zlatopolskiy et al. 2015; Brooks et al. 2014; Yusubov et al. 2013; Pike 2018). While these are an exciting class of precursor, not all structures are compatible with the chemistry required to produce them, reaction conditions for labelling are very harsh, and the precursors have a limited shelf life.

In addition to the iodonium salt precursor class, a new boronic acid pinacol ester precursor has become of significant interest for aryl radio fluorination, with the functional group being known for good long-term stability and chemical resilience (Preshlock et al. 2016; Mossine et al. 2015; Tredwell et al. 2014; Zarganes-Tzitzikas et al. 2019). The synthetic routes for these compounds are also well characterized due to their prevalence in synthetic chemistry, and the conditions for radiolabelling are not as harsh as those for iodonium salts.

Utilizing these two types of precursor, this paper presents the synthesis and automated radiolabelling of Oncrasin-like molecules for the purpose of development as PET imaging agents.

## Methods

### General

Solvents and reagents other than boron containing compounds were purchased from Sigma Aldrich and used without further purification. Boron compounds were purchased from Advanced Molecular Technologies and used without further purification.

#### Synthesis of compounds

##### Sodium hydride coupling reaction with benzyl halides

Sodium hydride (60%, 97 mg, 2.4 mmol) was washed with petroleum spirits (20 mL) twice before addition of a chilled solution of indole 3 carboxaldehyde (200 mg, 1.37 mmol) in THF (20 mL) to afford a pink coloured solution. The desired benzyl bromide (1.57 mmol) was added to the solution, with the ice bath being removed 5 min after this addition. This reaction mixture was stirred at room temperature overnight before termination of the reaction by removal of solvent under vacuum. The resultant residue was then redissolved in 30 mL of diethyl ether and washed with saturated sodium bicarbonate (2 × 50 mL) and distilled water (2 × 50 mL). The organic layer was then dried onto silica for column chromatography. Fractions were combined and concentrated under reduced pressure to afford the product. Crystals were grown either through evaporation from dicholormethane or vapour diffusion methods from either ethyl acetate, ethanol, or methanol with petroleum spirits.

##### Miyarua borylation

A flask was charged with N-(4-iodobenzyl)-indole-3-carbaldehyde (500 mg, 1.38 mmol), potassium acetate (407 mg, 4.14 mmol), bispinacolato diborane (1.75 g, 6.9 mmol) and palladium dichloride diphenyl phosphine ferrocene catalyst (30 mg, 0.04 mmol). The mixture was dissolved in 20 mL of DMF and heated to 70 °C for 3 h before being diluted into 300 mL of water and extracted 3 times with dichloromethane. The organics were combined and then dried onto silica for column chromatography. Fractions were combined and concentrated under reduced pressure to yield a mass of 360 mg (72%).

#### Radiochemistry

##### Production of fluoride

No-carrier-added [^18^F] fluoride was produced by the ^18^O(p,n)^18^F nuclear reaction with an 10 MeV proton beam generated by the IBA Cyclone 10/5 cyclotron in a titanium target using [^18^O] H_2_O at Austin Health, Department of Molecular Imaging and Therapy. Typical irradiation parameters were 16 μA for 30 min, which resulted in 10.4–11.8 GBq (281–318 mCi) of [^18^F] fluoride being transferred into the synthesis module. These yields are lower than the theoretical yield and are explained by the use of recycled [^18^O]H_2_O of unknown isotopic enrichment.

The [^18^F]F- ion was isolated from [^18^O]H_2_O by trapping on a QMA ion exchange column (light sepPak cartridge, Waters),which was preconditioned with 0.5 M K_2_CO_3_ solution followed by washing with distilled water. The [^18^F]F- ion was eluted off the column into a reactor containing 1 mL of anhydrous acetonitrile using a solution containing either a standard potassium carbonate eluent or a potassium triflate eluent. Following elution, repeated azeotropic evaporation with acetonitrile (2 × 1 mL) to dryness was undertaken to give the anhydrous [18F] fluoride ion used in the radiolabelling experiments.

##### Preparation of copper catalyst

Fifteen milligram (4.1 μm) of Copper(II) triflate was dissolved in 400 μL of DMF and combined with 100 uL of a 50:50 mixture of DMF and pyridine. This mixture was sonicated to ensure proper solvation of the copper salt and added to the catalyst vial in the FlexLab as described in Table 1. *Preparation of eluents.*

**Table 1:** Flexlab set up for radiolabelling reactions

Container	Reagents
Vial 1	Eluent
Vial 2	4 mg of precursor in 500 μl of DMF
Vial 3	1 ml anhydrous acetonitrile
Vial 4	450 μl of DMF. 50 μl of Pyridine, 15 mg of Cu(OTf)_2_
Vial 5	1 ml of Acetonitrile and 1 ml of distilled water
Vial 6	
Vial 7	
Vial 8	
Vial 9	
Vial 10	
Vial 11	10 ml of distilled water
Vial 12	1 ml of ethanol
Vial 13	1 ml of saline
Vial 14	
Vial 15	
Vial 16	
Vial 17	
Vial 18	
Vial 19	
QMA seppak	Quaternary Methyl Ammonium Cartridge
Seppak A	
Seppak B	
Seppak C	
Seppak D	C18 seppak
HPLC Vial 1	
HPLC Vial 2	40 ml of distilled water
Reactor 1	1 ml anhydrous acetonitrile
Reactor 2	
Loop Vial 1	
Loop Vial 2	1 ml distilled water
HPLC eluent A	0.1% Ammonium formate
HPLC eluent B	Acetonitrile
HPLC eluent C	Water
HPLC eluent D	Ethanol

For preparation of carbonate eluent, 4 mg (28.9 μm) of potassium carbonate and 12 mg (3.1 μm) of kryptofix 2.2.2 was solvated in 300 μL of water and 700 μL of acetonitrile.

For preparation of bicarbonate eluent, 2 mg (20 μm) of KHCO_3_ was combined with 11 mg (2.9 μm) of kryptofix 2.2.2. and dissolved in 1 mL of methanol and 200 μL of water.

For preparation of tetrabutyl ammonium fluoride, 300 μL of a 50 mM tetrabutylammonium bicarbonate solution in 5% ethanol was added to 600 μL of acetonitrile.

Potassium triflate eluent was prepared by the solvation of 2.3 mg (12.2 μm) of potassium triflate was dissolved in 500 μL of distilled water and 500 μL of acetonitrile to which 9 mg (2.4 μm) of kryptofix 2.2.2. was added. The mixture was sonicated to ensure proper solvation and mixing of components and added to the eluent vial of the Flexlab module as described in Table 1.

##### Radiolabelling

**Fig. 2:**
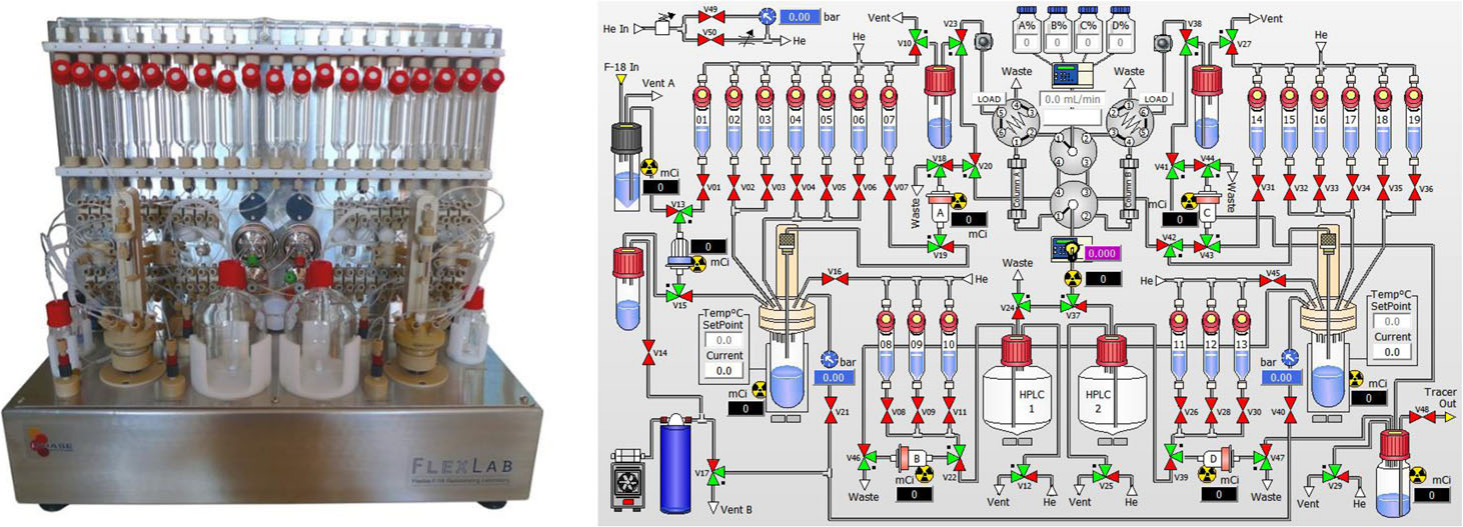
Flexlab module (left) and interactive control interface (right)

Radiolabelling reactions were undertaken using an iPhase Flexlab automated synthesis module. This module is equipped with multiple reaction vessels and two HPLCs, allowing the user to undertake complex, multistep radiosynthetic reactions. Depictions of the module and interactive interface used to control it can be found Fig. 2.

Four milligram of the appropriate radiotracer precursor was dissolved in 0.5 mL of DMF and combined with the previously described catalyst system with a kryptofix chelated ^18^F fluoride source. The reaction was heated to 120 °C for 20 min, with purging of the reactor every 5 min with atmospheric air for 3 s. After this time, the reaction was cooled to room temperature before dilution with 1 mL of water and transfer to a loop vial in preparation for HPLC. The reaction vessel was then washed with a further 2 mL of 50:50 H_2_O:ACN to ensure full recovery of the reaction mixture before subsequent HPLC purification of the combined fractions. After initial injection of the reaction mixture, the HPLC was run for 2 min with a 0.1% ammonium formate in H_2_O solution before switching to a gradient starting at 80:20 0.1% ammonium formate in H_2_O:ACN progressing to 10:90 over 18 min. Products were collected between 17 and 23 min depending on the tracer. Products were trapped using a preconditioned C18 SPE cartridge. This cartridge was conditioned with 1 mL of ethanol followed by 10 mL of water and then drying before use. For elution of the product, 1 mL of ethanol was used, with an additional 1 mL of distilled water being used to rinse the cartridge and tubing into the product vial.

## Results

### Synthesis of cold standards

**Scheme 1:**
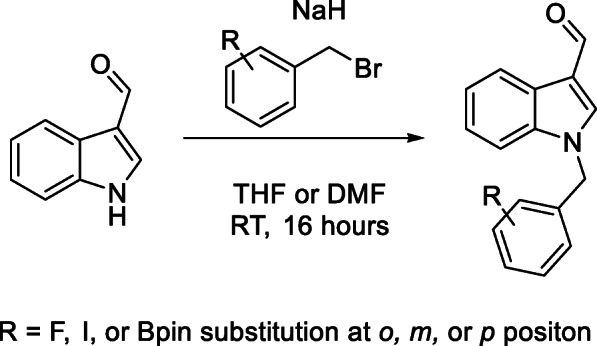
Standard coupling conditions for indole-3-carbaldehyde and various benzyl bromides

The synthesis of cold standards was carried out for spectroscopic characterization and identification, as well as a comparison with radiolabel products for confirmation of product by radio HPLC. Synthetic procedures were undertaken as outlined in Scheme 1 to produce cold standards in a good yield. Testing of potency relative to the parental Oncrasin-1 compound was undertaken, with all 3 fluorinated compounds demonstrating superior selectivity between sensitive cells and insensitive cells, with results shown in the SI.

#### Synthesis of precursors

##### Coupling of indole-3-carbaldehyde and benzyl halides

A general sodium hydride deprotonation of the indole followed by addition of a benzyl halide of the appropriate substituent and substitution pattern was used for generation of cold standards as well as precursors and iodinated intermediates, as shown in Scheme 1. Yields for these compounds were typically above 70%, with the *ortho* substituted boronic acid pinacol ester derivative yielding lower at 58%.

##### Miyarua borylation

A standard Miyaura borylation was utilized for generation of the parasubstituted boronic acid pinacol ester precursor from an iodine substituted intermediate to afford the product in a 72% yield.

##### Iodonium salt precursors

**Scheme 2:**
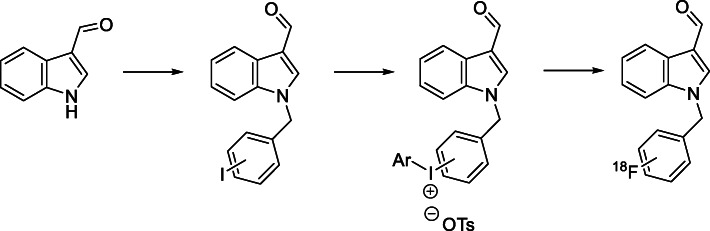
Proposed synthetic route for iodonium salt synthesis and subsequent labelling

**Scheme 3:**
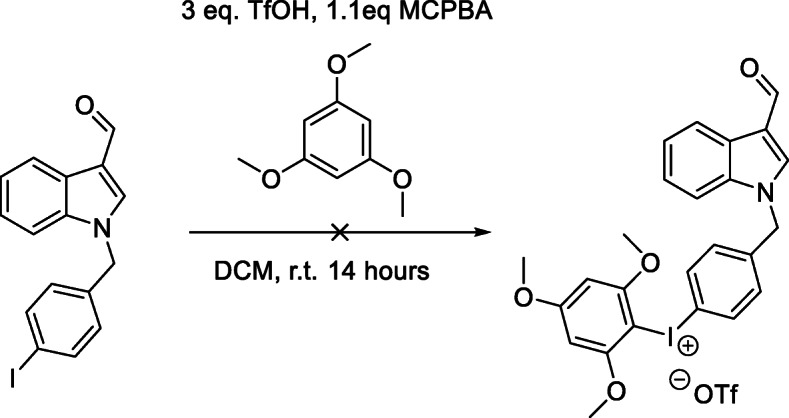
Attempted initial iodonium salt synthesis

The initial synthetic route to an iodonium salt precursor was envisioned as shown in Scheme 2,with a sodium hydride coupling between commercially available indole-3-carbaldehyde and the appropriately substituted iodo benzyl bromide. This intermediate would then undergo a one-pot reaction to form an iodonium salt suitable for radiolabelling. In order to determine optimal conditions, initial reactions were carried out with the *para* substituted material, as shown in Scheme 3.

*Synthesis of of 1-(4-iodo-benzyl)-1H-indole-3-carbaldehyde (Compound 1)* was undertaken as described in Scheme 1 and the product was obtained in quantitative yield. With Compound 1 in hand, a one-pot synthesis of the iodonium salt was trialled using reaction conditions described by Zhu, Jalalian and Olofsson (Zhu et al. 2008), shown in Scheme 3. The resultant reaction mixture showed no indication of product formation. The characteristic proton signal correlating to the aldehyde proton at approximately 10 ppm was absent and the expected mass for the desired product was not detected with high resolution mass spectrometry, suggesting an incompatibility between these reaction conditions and the aldehyde functional group. Literature indicated that sodium periodate (Kazmierczak et al. 2001) and peroxide (Merritt and Olofsson 2009) could also be employed as oxidants and were trialled with similar results. A summary of reactants attempted with Compound 1 shown in Table 2 with none of the combinations resulting in any identifiable products other than starting material.

**Table 2:** Reaction conditions trialled with for iodonium salt synthesis

Oxidant	Counter ion	Aryl System
MCPBA	OTf/OTs	*p*-Methoxybenzene (Bielawski et al. 2007)
Peroxide	OTs	1,3, Dimethoxy Benzene
Oxone	OTs	1,3,5 Trimethoxy Benzene
Sodium Periodate (Kazmierczak et al. 2001)	Acetic acid/H_2_SO_4_	1,3,5 Trimethoxy Benzene

**Scheme 4:**
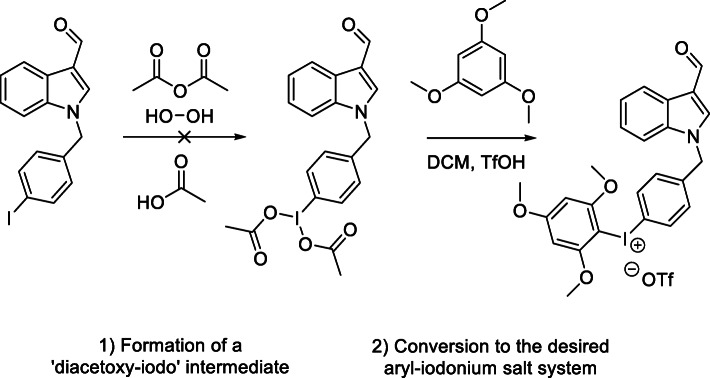
A 2-step approach to iodonium salt synthesis

**Fig. 3:**
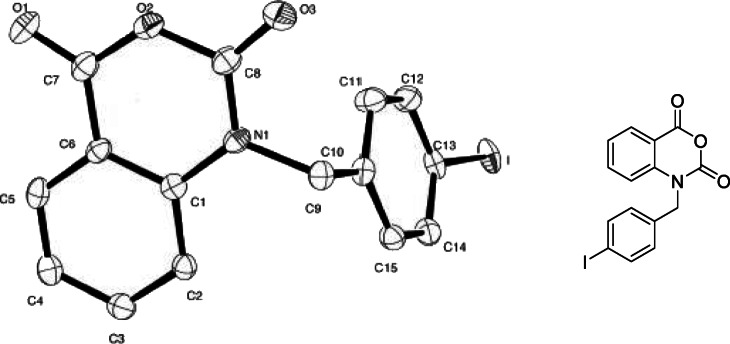
Crystal structure and chemical drawing of a major product Step 1 in Scheme 4

Attempts to carry out iodonium salt forming reactions in a two-step process (Chun and Pike 2012) shown in Scheme 4 also yielded no identifiable compounds. Being unable to isolate a satisfactory salt from these conditions, a major product from the oxidation step of the two-step reaction was isolated via column chromatography and crystallized using vapour diffusion methods for x-ray crystallography, with the structure shown in Fig. 3.

##### Boronic acid pinacol ester precursors

**Scheme 5:**
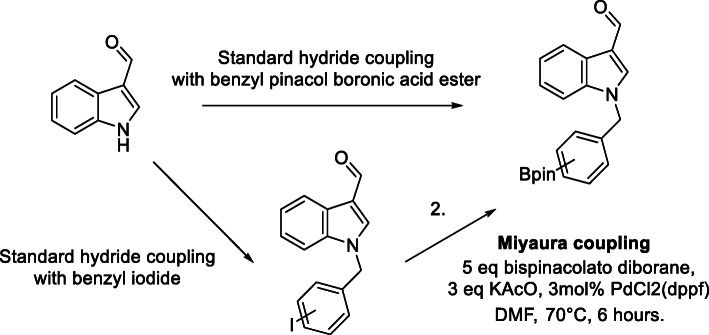
Synthesis of boronic ester precursors via two routes

With an iodine containing intermediate already in hand (Compound 1), a Miyarua borylation reaction was undertaken to provide the *para* substituted precursor in a 72% yield, shown in Scheme 5. As previous coupling reactions had been undertaken successfully, commercially available sources of para*, meta* or *ortho* substituted boronic acid pinacol ester containing benzyl bromides were used to investigate a one-step coupling synthetic route. This method produced the desired product in yields 78%, 76% and 58% yields of the *para*, *meta*, and *ortho* precursors respectively. Product identity was confirmed through spectroscopy as well as x-ray crystal structures.

With three precursors in hand, radiolabelling experiments were undertaken.

##### Radiochemistry

**Scheme 6:**
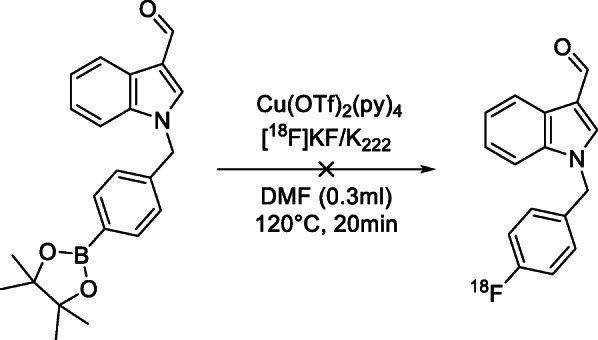
Initial radiolabelling trial conditions utilizing Treadwell conditions

Reaction conditions adapted from Tredwell et al. (Tredwell et al. 2014) were utilized for initial radiolabelling experiments, as shown in Scheme 6. Under these conditions, no radiolabelled products were isolated.

As initial radiolabelling attempts yielded no discernible radiolabel products and previous literature suggested that these reactions may not be suitable for automated synthesis (Tredwell et al. 2014), possibly due to the inert gas systems they often operate under; the mechanism of catalysis for these reactions is unclear but may operate through a Cham-Lam coupling-like oxidation cycle (Vantourout et al. 2017), which would require atmospheric oxygen that is not present in standard, inert gas flushed automated systems. The reaction was attempted again with air being purged into the reaction vessel throughout the labelling, with no improvement in radiolabel incorporation.

A 4-Methoxycarbonylphenylboronic acid, pinacol ester was utilized as a model for trouble shooting as it is chemically similar to reagents used in both the Tredwell paper and another paper authored by Mossine et al. (Mossine et al. 2015), which had resulted in excellent yields, however under the previously stated conditions, no radiolabelling was observed.

**Scheme 7:**
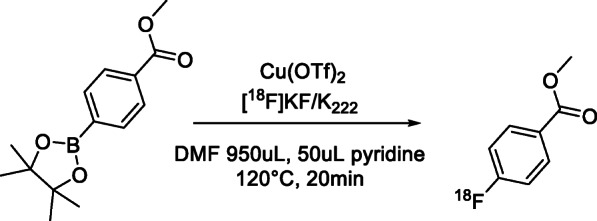
Radiolabelling conditions used for labelling of model system

As the system described by Tredwell was not able to produce radiolabelled products in our hands, another similar system, described by Mossine et al. and shown in Scheme 7 was investigated.

This system also produced no discernible radiolabelled products at the expected retention time. Mossine and co-workers had noted poor radiochemical yields prior to their own optimization for boronic acid labelling with regards to eluents. Development of an alternate eluent was required for successful synthesis, which utilized a minimized quantity of potassium carbonate and potassium triflate in combination with 2.2.2 kryptofix.

Attempts to carry out the radiosynthesis with the model system utilizing other standard eluents such as bicarbonate and tertbutyl amine were unsuccessful.

To determine if the eluent was the limiting factor in the radiosynthesis, a synthesis was carried out without a QMA cartridge facilitated fluoride isolation, with evaporation of the [18F] fluoride containing ^18^O water being performed in the absence of additives such as kryptofix prior to the labelling reaction. This system yielded small amounts of previously unobserved radiolabelling products. Adoption of a potassium triflate eluent system afforded radiolabelling of the model system as the major radiolabel product. Further optimization of the eluent showed that the preconditioned QMA cartridge used for an ^18^F-FDG synthesis contained enough bicarbonate for labelling and so this was removed from the eluent. When using QMA cartridges which had been reconditioned after initial use, significant variability was observed, so this was avoided for future synthesis. A summary of conditions trialled with the model compound is shown in Table 3.

**Table 3:** Trialled reaction conditions for model systems

Reaction	1	2	3	4	5
Eluent	Carbonate	Tetrabutylamine	Bicarbonate	**None**	Triflate
Catalyst	Tetrakis Complex	Tetrakis Complex	Tetrakis Complex	Cu(OTf)_2_ + Pyridine	Cu(OTf)_2_ + Pyridine
Kryptofix	10 mg	–	4.5 mg		4.5 mg
Outcome	Nil	Nil	Nil	Minor Product	Product

**Scheme 8:**
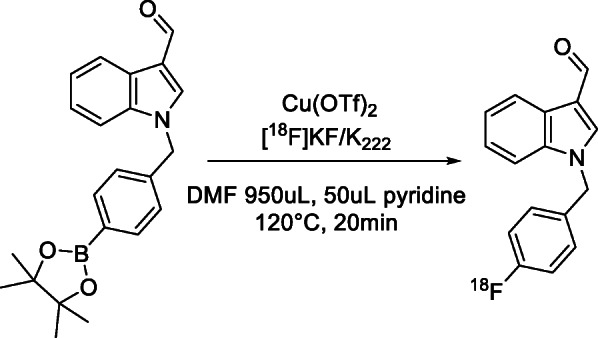
Radiolabelling conditions for KAM001

**Fig. 4:**
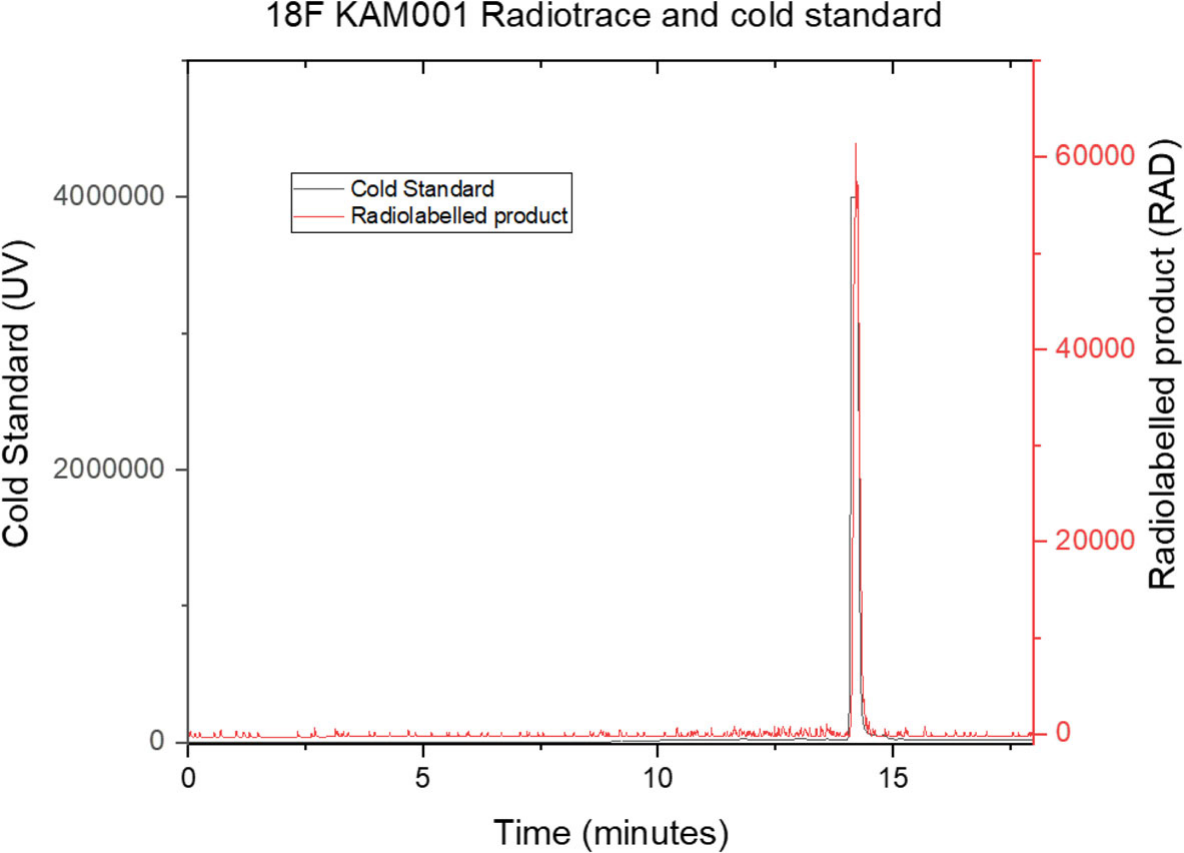
^18^F KAM001 identity confirmation using cold standard retention

**Fig. 5:**
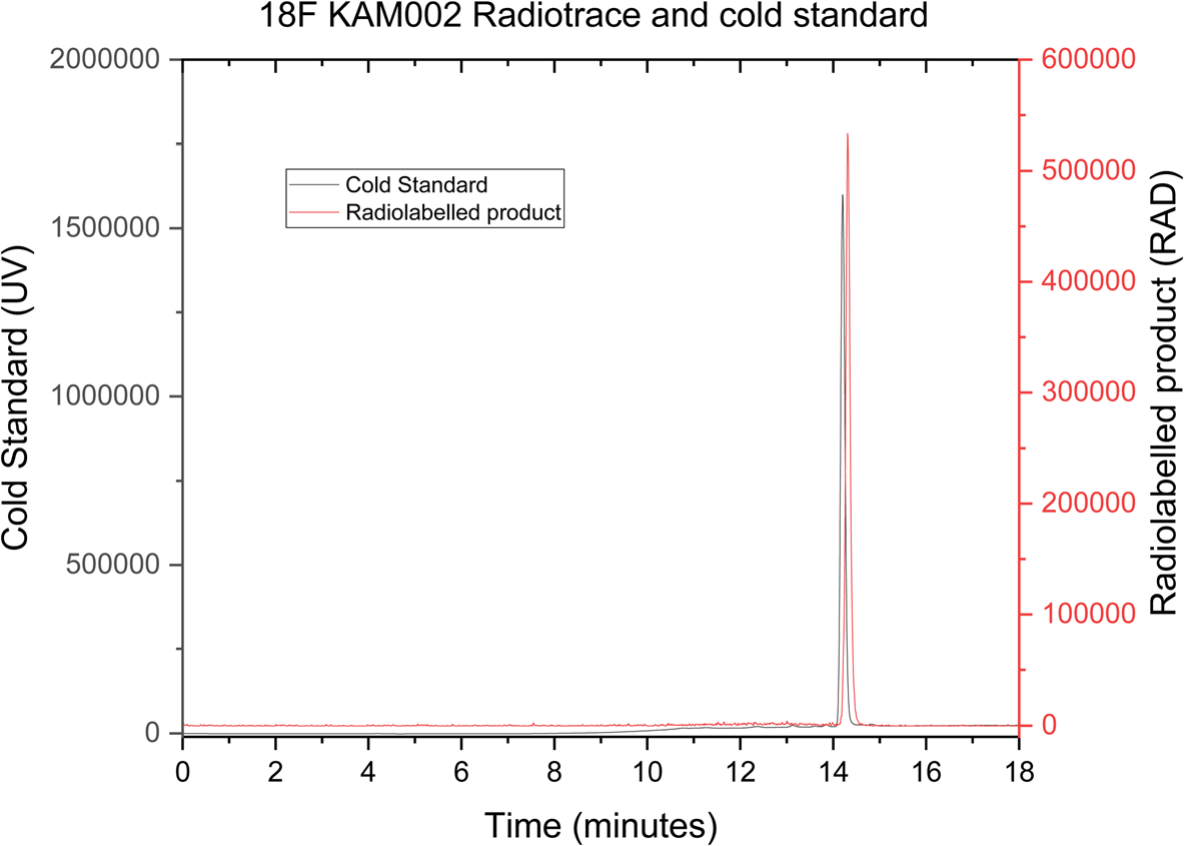
^18^F KAM002 identity confirmation using cold standard retention

**Fig. 6:**
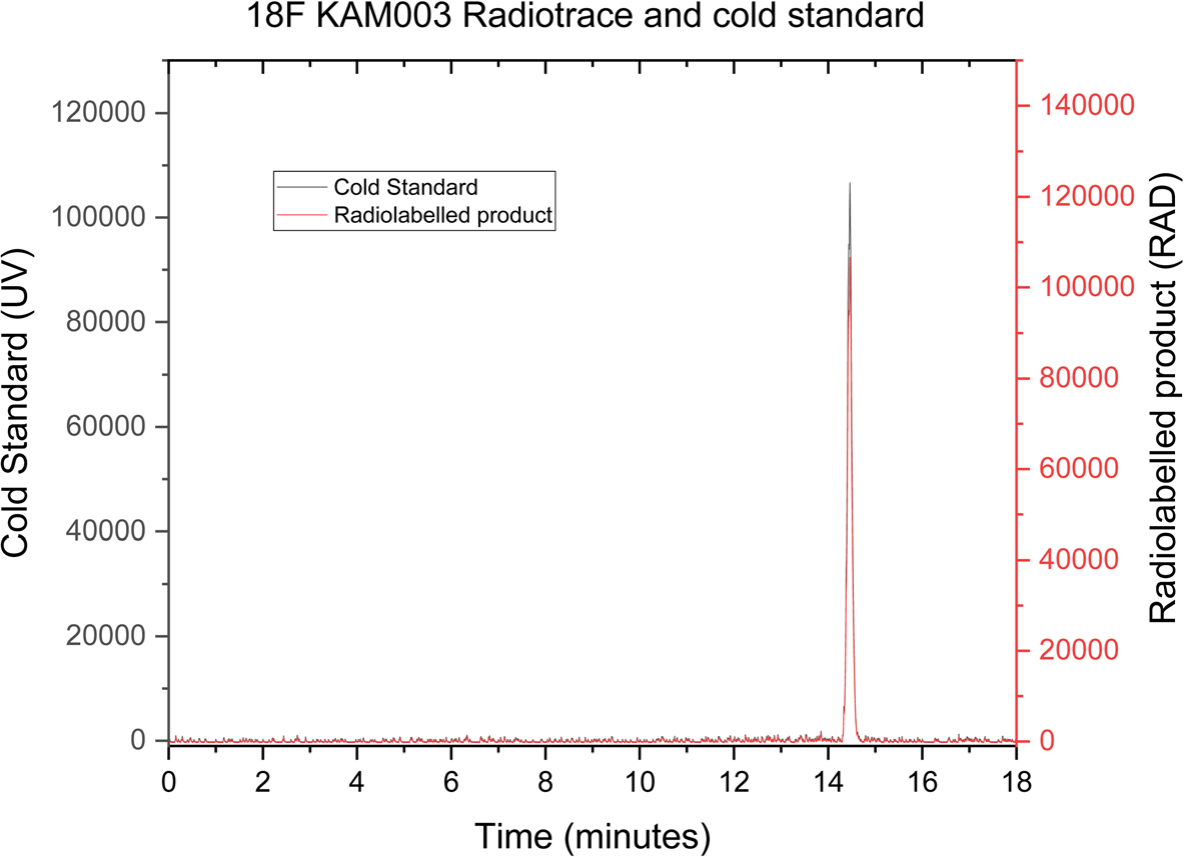
^18^F KAM003 identity confirmation using cold standard retention

Having successfully produced a radiolabelled molecule in the model system, the BpinKAM001 system was revisited, utilizing the revised catalyst system and new eluent, with successful product formation being achieved through use of the conditions shown in Scheme 8. HPLC purification of the radio peak from the reaction mixture was undertaken in the Flexlab module, with a representative trace shown in the supplementary information, and confirmed to be the desired radiotracer by registration with the cold standard peak retention time, as shown in Fig. 4. Using these conditions radiolabelling of the remaining BpinKAM002 and BpinKAM003 compounds was undertaken successfully, with HPLC traces of purified products shown Figs. 5 and 6.

Decay corrected yields for the purified tracers were 10.76% ± 0.96% (*n* = 5), 14.7% ±8.58% (*n* = 3) and 14.92% ±3.9% (n = 3) for ^18^F KAM001, ^18^F KAM002 and ^18^F KAM003 respectively. All tracers were shown to have a radiochemical purity greater than 99%, with a representative run of the 18F KAM002 producing a molar activity of 1.09 GBq/μmol.

## Discussion

### Synthesis of iodonium salt precursors

Initial reactions to form iodonium salts were planned as described in Scheme 2; A standard sodium hydride coupling would be the first step to form the benzyl iodide. This compound could then be reacted to form an iodonium salt under various conditions using electron rich aryl systems denoted ‘Ar’. The iodonium salt would then undergo radiolabelling.

Reactions to attempt to form an iodonium salt precursor were incompatible with the indole scaffold contained in the Oncrasin molecule as the indole structure is preferentially being oxidized rather than the iodine in the benzyl ring. This process likely occurs with all of the oxidants utilized in the reactions undertaken, with a wide range of intermediates being produced, resulting in complex mixtures of products. The structure obtained from the peracetic acid reaction suggests a multi-step Baeyer–Villiger oxidation, to form isatin. This structure can then undergo a ring expanding reaction to form the isatoic acid structure shown in Fig. 3. These chemical transformations are consistent with the literature on the oxidation of indoles. While the trialled conditions were unable to produce the desired iodonium salt, Rotstein and coworkers have been able to access an indole iodonium salt through use of a sodium perborate oxidant (Rotstein et al. 2014), and oxidation at the ‘3 position to form iodonium salts has also been shown in literature (Moriyama et al. 2015). The difference between these works and labelling of these Oncrasin-like molecules is that we are attempting to form an iodonium salt on the benzyl group rather than the indole, which seems to be preferentially oxidized.

This understanding of indole sensitivity to the oxidation required for iodonium salt forming reactions is a significant development for ^18^F radiochemistry, as indoles are an important biological scaffold and are present in many structures which may be investigated as PET imaging agents including amino acids, peptides and small molecule drugs.

As the indole in the Oncrasin structure is not amenable to the trialled oxidation conditions for a direct iodonium salt synthesis, attention was directed towards the boronic acid pinacol ester precursors.

Pinacol boronic esters precursors were readily produced through two synthetic routes, with good yields for both routes, proving to be a superior precursor class for this indole containing compound.

The lower yield of the *ortho* product can be explained by a steric clash observed in the crystal structure of this product, which would make nucleophilic attack of the halogen less favoured, hindering product formation.

### Radiochemistry

Initial experiments trialling both Tredwell and Mossine catalyst systems were unsuccessful due to use of an eluent system which was too basic for the copper catalyst system. This was identified as a potential problem for the catalyst system in Mossine’s work (Mossine et al. 2015) and since this work significant efforts have been directed optimizing eluents for these copper catalysed systems (Mossine et al. 2017). The bicarbonate in the eluent was likely able to displace the triflate and pyridine molecules co-ordinated with the copper, inactivating the catalyst.

Trialling the reaction of the boronic esters without the eluent system helped to identify this as the confounding factor for this chemistry, and this finding is consistent with other literature around these methods (Mossine et al. 2017).

Yields for these Oncrasin-like molecules are somewhat low compared to other literature report for benzyl labelling but in a similar range to labelling of indoles. Both Mossine and Tredwell papers noted that precursors containing nitrogen produced lower radiochemical yields when utilizing the copper catalysed labelling. This lower yield could potentially be due to the precursor competing with pyridine in forming the catalyst system, both deactivating the catalyst and sequestering precursor so that it is not available for radiofluorination.

Recent work by Zarganes-Tzitzikas et al. (Zarganes-Tzitzikas et al. 2019) suggest that increasing the quantities of precursor approximately 4 fold could improve radiolabelling yields, however protodeborylation side products which were observed were difficult to separate and would likely be increased with the increase in precursor. This would potentially impact on the purity and molar activity of the tracer. Compared to other reported radiofluorination reactions using this system, the molar activity of the current synthesis is relatively low (Zlatopolskiy et al. 2015) and is likely due to the coelution of these side products Protodeborylation side products may have an effect on the imaging characteristics of the compound, with the unfluorinated structure showing activity, albeit significantly decreased activity, in the literature (Wu et al. 2011). The quantity of tracer produced was sufficient for preliminary biological evaluation of the molecules. For translation to the clinic, production scale up would likely be required and further optimization of radiotracer and by-product separation would be ideal for higher molar activities.

## Conclusions

Synthesis of Oncrasin-like molecules was attempted through both an iodonium salt precursor and a boronic acid pinacol ester precursor. Reaction conditions for direct synthesis of an iodonium salt precursor were not compatible with the indole scaffold in the structure. Boronic acid pinacol ester precursors were successfully synthesized and radiolabelled utilizing modified reaction conditions reported by Mossine and co-workers. Identifying the incompatibility of the indole scaffold and common iodonium salt-forming reaction conditions is an important contribution to field of radiotracer development. In our hands these reaction conditions seem to favour oxidation of the indole scaffold over the desired benzyl iodide in the target precursors. Synthesis of these radiotracers will allow for further work to determine suitability of Oncrasin-based molecules as potential diagnostics for cancer detection and tumour type differentiation.

## Supplementary Information

**Additional file 1.** Supplementary Data.

## Data Availability

Crystal structure generated and/or analysed during this work are available in the Cambridge Crystal Structure Database repository. https://www.ccdc.cam.ac.uk/ All other data generated or analyzed during this study are included in this published article and its supplementary information files.

## Abbreviations

Ar: Aryl group
RCY: Radio chemical yield
RAS: Rat Sarcoma
KRAS: Kirsten Rat Sarcoma
EGFR: Epidermal Growth Factor Receptor
PET: Positron Emission Tomography
DMF: Dimethylformamide
ACN: Acetonitrile
HPLC: High Performance Liquid Chromatography
GBq: Giga-becquerel
SPE: Solid Phase Extraction
MCPBA: *Meta*-chloro perbenzoic acid
OTf: Triflate anion
OTs: Tosylate anion
QMA: Quaternary Methyl Ammonium
FDG: Fluorodeoxy glucose
