# Supplementary material for: Automated synthesis of 18F radiolabelled indole containing Oncrasin-like molecules; a comparison of iodonium salts and boronic ester chemistry
Source: EJNMMI Radiopharm Chem. 2020 Nov 9;5:23. doi: 10.1186/s41181-020-00104-x (PMC7652984; doi:10.1186/s41181-020-00104-x)
Supplement: Supplementary file 1 — Additional file 1. Supplementary Data. [file 41181_2020_104_MOESM1_ESM.docx]

**Supplementary data**

*General*

Solvents and reagents other than boron containing compounds were purchased from Sigma Aldrich and used without further purification. Boron compounds were purchased from Advanced Molecular Technologies and used without further purification.

*NMR experiments*

Nuclear magnetic resonance (NMR) spectra for ^1^H and ^13^C nuclei were recorded using a Varian Inova-600 NMR operating at 600 and 150 MHz respectively. Unless otherwise specified, 1H NMR were obtained in deuterochloroform solution (CDCl_3_) at ambient temperature with residual chloroform of δ7.26 ppm as internal reference. ^1^H NMR chemical shifts are reported in parts per million (ppm) and are followed by integration, multiplicity (singlet (s), doublet (d), triplet (t), multiplet (m), doublet doublet (dd), triplet doublet (td), broad singlet (b.s.)), coupling constant (J) given in Hertz (Hz). Proton decoupled ^13^C NMR chemical shifts were referenced on the centre peak of CDCl_3_ (δ77.0 ppm) and the signals are reported as chemical shift in ppm.

*Mass Spectrometry*

High Resolution Mass Spectrometry (HRMS) was carried out in the positive ion mode on either a Finnigan LTQ-FT hybrid linear ion trap fitted with an electrospray ionization (ESI) source and Fourier Transform Ion Cyclotron Resonance (FT-ICR) or a Thermo NanoLC/ OrbiTRAP ELITE ETD mass spectrometer. All values are represented in a mass to charge ratio (m/z).

**Characterization of compounds**

**Structure**

**Identifier - KAM001**

**Mass Spec**

Chemical Formula: C16H12FNO

Exact Mass: 253.09029

Molecular Weight: 253.27098

Calculated Mass (M +H) 254.09757

Observed Mass (M +H) 254.09747

**^1^H NMR** (CDCl_3_, 600 MHz) δ 5.34 (s, 2 H), 7.05 (t, J = 8.64 Hz, 2 H), 7.17 (dd, J = 8.57, 5.20 Hz, 2 H), 7.29 - 7.37 (m, 3 H), 7.71 (s, 1 H), 8.27 - 8.42 (m, 1 H), 10.02 (s, 1 H)

**^13^C NMR:** CDCl_3_, 600 MHz 50.3, 110.2, 116.1, 116.2, 122.2, 123.1, 124.2, 128.9, 129.0, 138.1, 184.5

**Melting Point 1**14.1-116.3 °C

**High Resolution Mass Spectrometry of KAM001**


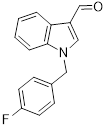

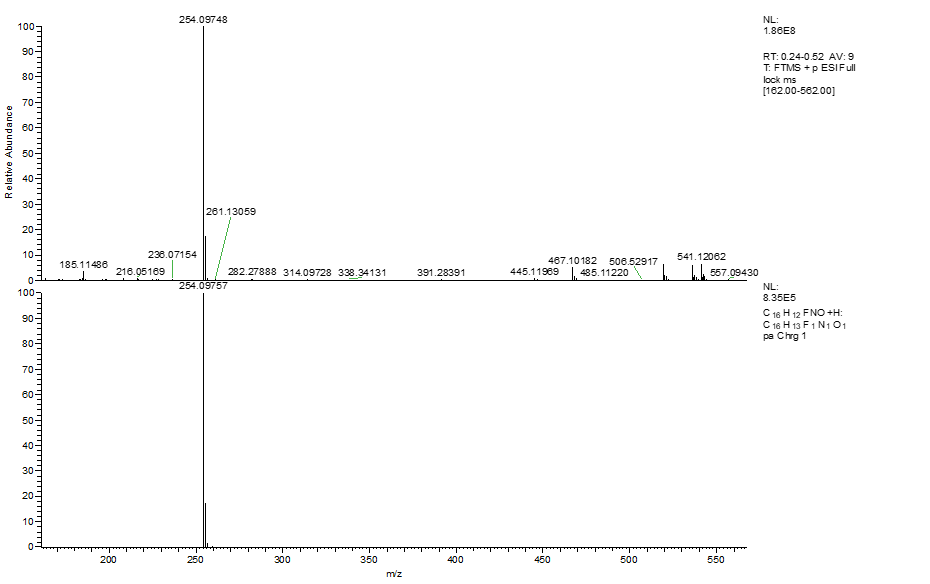


**^1^H NMR of KAM001**


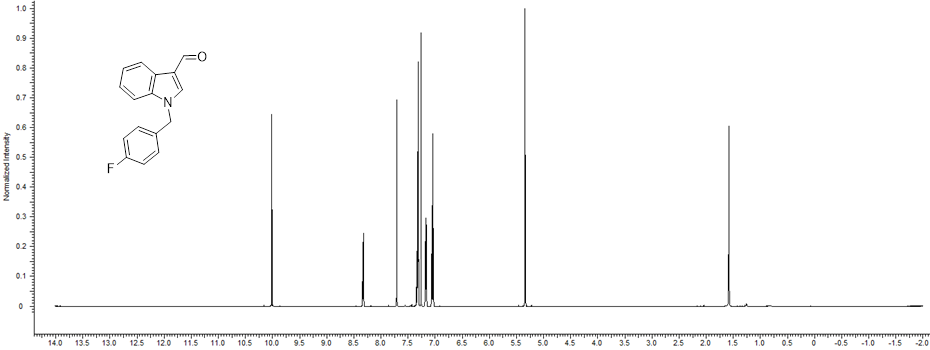


**^13^C NMR of KAM001**


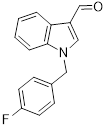

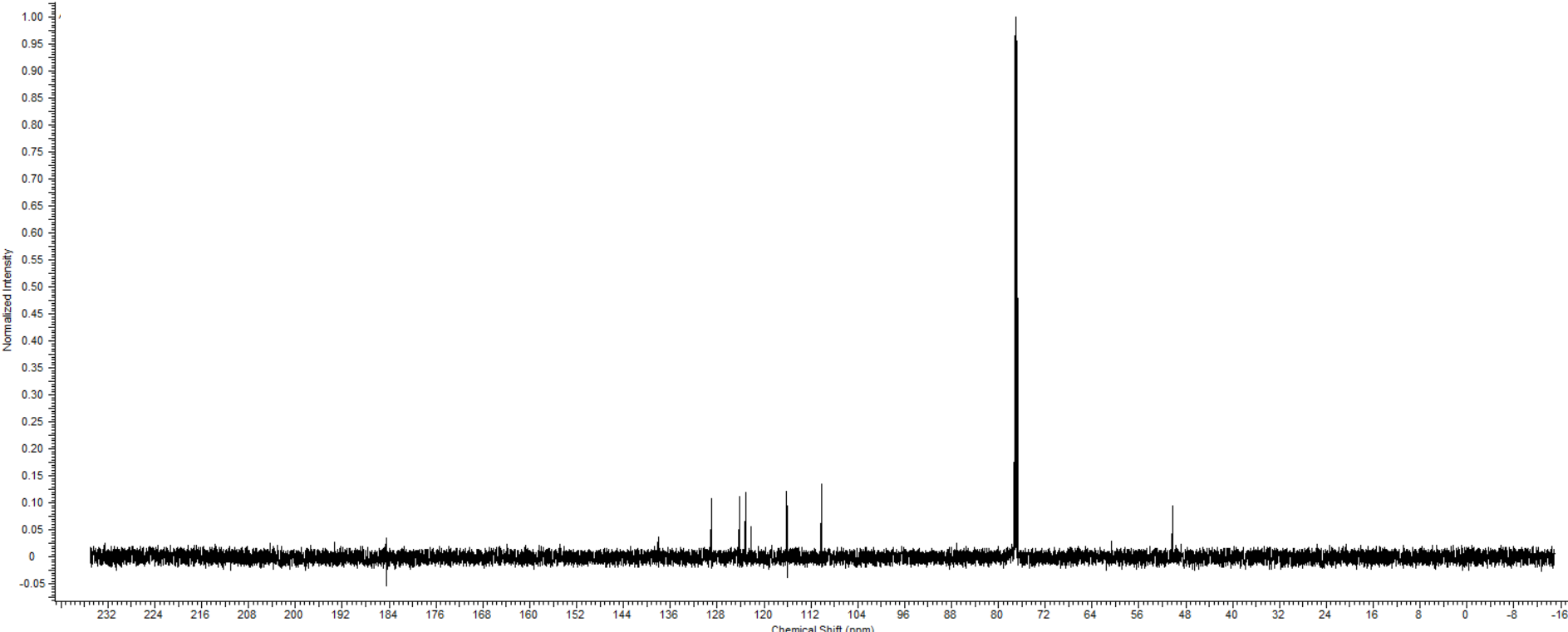


**Structure**

**Identifier - KAM002**

**Mass Spec**

Chemical Formula: C16H12FNO

Exact Mass: 253.09029

Molecular Weight: 253.27098

Calculated Mass (M +H) 254.09757

Observed Mass (M +H) 254.09747

**^1^H NMR** (CDCl_3_, 600 MHz) δ 10.03 (s, 1H), 8.15 - 8.55 (m, 1H), 7.74 (s, 1H), 7.23 - 7.45 (m, 5H), 6.70 - 7.12 (m, 3H), 5.37 (s, 2H)

**^13^C NMR: δ**: 50.4, 110.2, 114.0, 114.1, 115.3, 115.5, 118.7, 122.2, 122.6, 123.2, 124.3, 125.5, 130.7, 130.8, 137.3, 137.9, 138.3, 162.3, 163.9, 184.6

**Melting Point** 109.4-112.3 °C

Crystal Structure

Link: DOI: 10.5517/ccdc.csd.cc23sy95


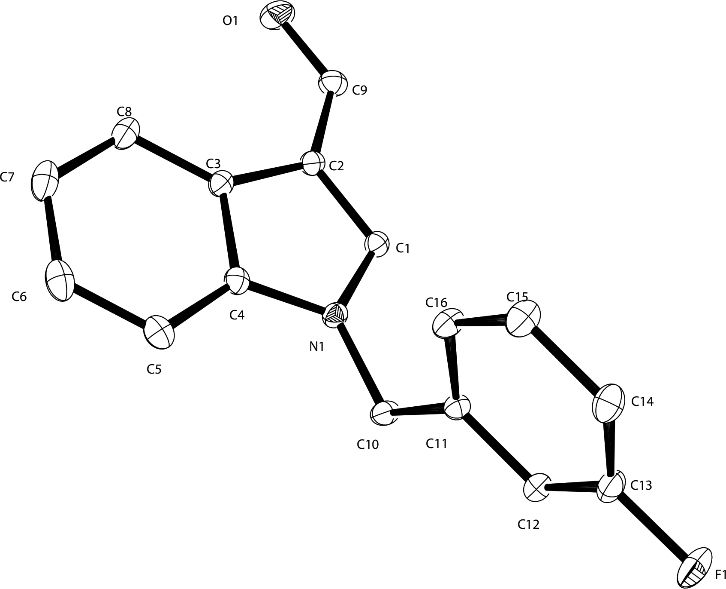


**High Resolution Mass Spectrometry of KAM002**


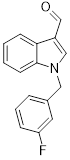

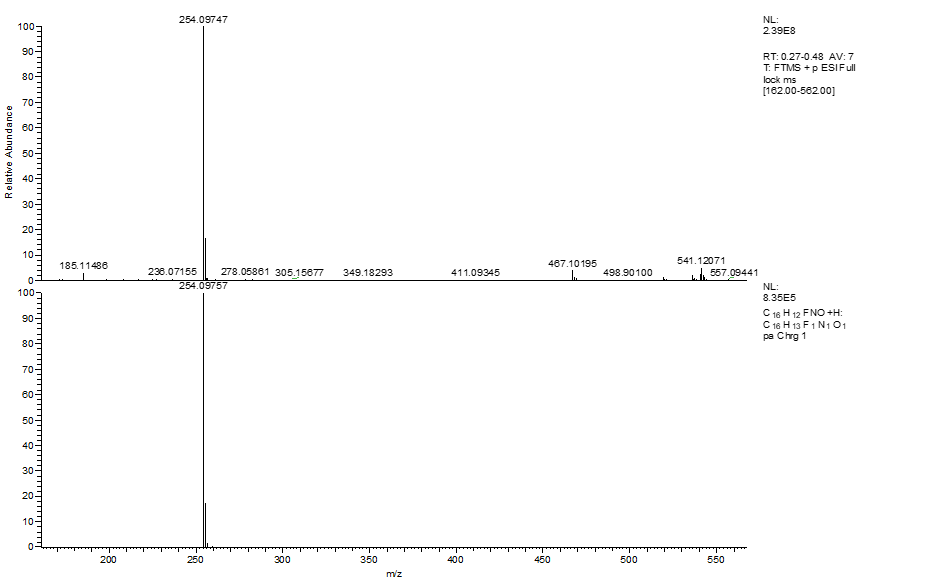


**^1^H NMR of KAM002**


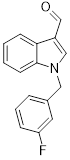

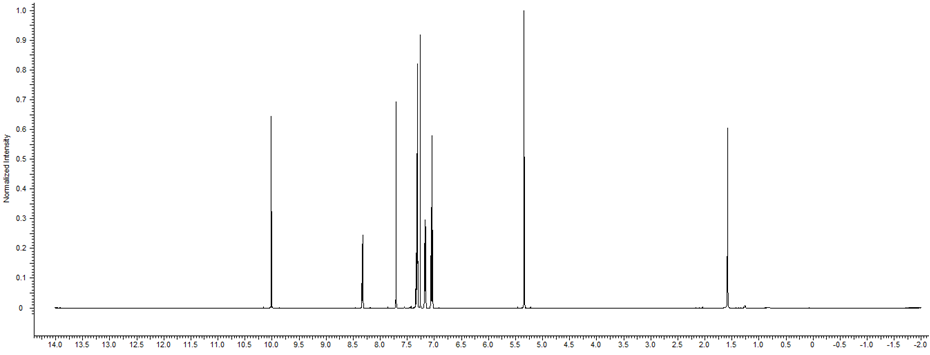


**^13^C NMR of KAM002**


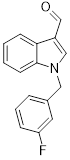

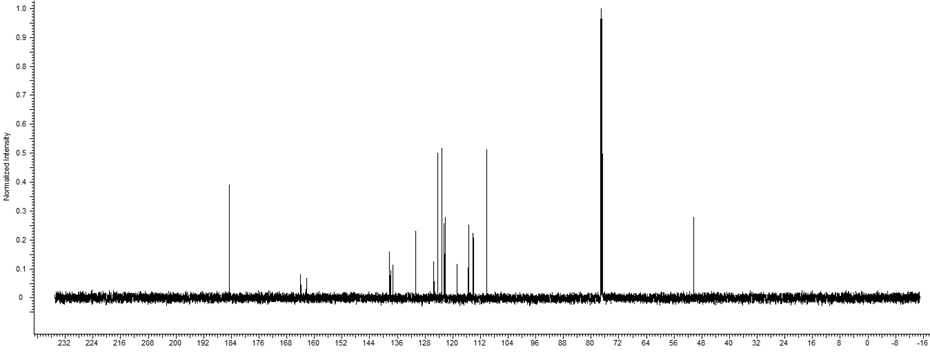


**Structure**

**Identifier - KAM003**

**Mass Spec**

Chemical Formula: C16H12FNO

Exact Mass: 253.09029

Molecular Weight: 253.27098

Calculated Mass (M + H) 254.09029

Observed Mass (M + H ) 254.09753

**^1^H NMR**

(CDCl_3_, 400 MHz) δ 9.88 - 10.08 (m, 1H), 8.20 - 8.45 (m, 1H), 7.77 (s, 1H), 7.36 - 7.44 (m, 1H), 7.28 - 7.36 (m, 3H), 7.00 - 7.18 (m, 3H), 5.41 (s, 2H)

**^13^C NMR: δ**:

**Melting Point** 98.0-100.5°C

Crystal Structure

Link: https://dx.doi.org/10.5517/ccdc.csd.cc23syb6


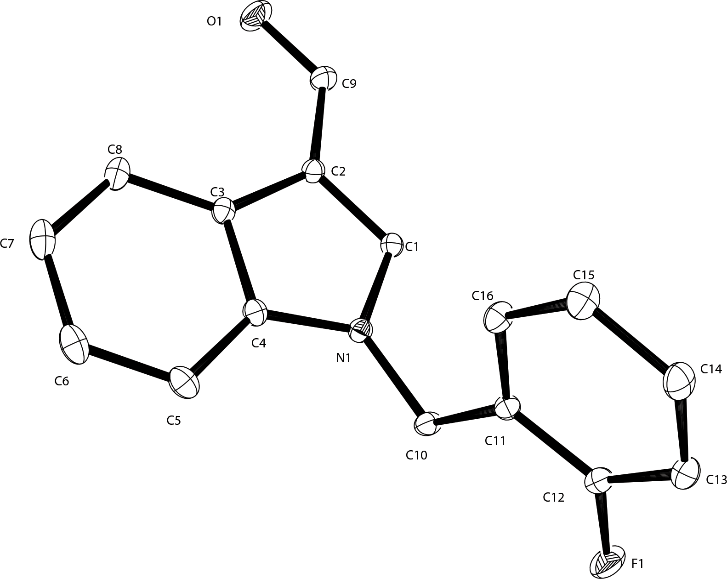


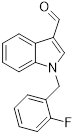

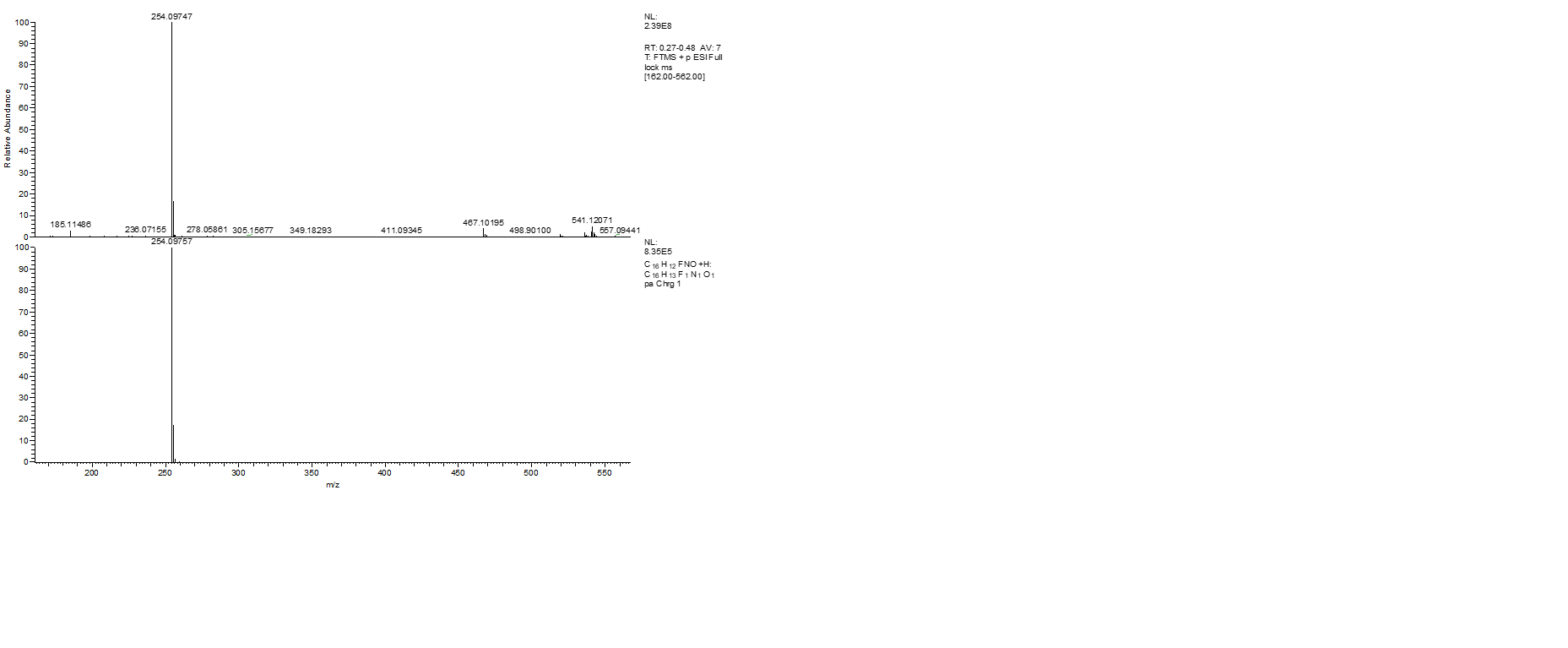


**^1^H NMR of KAM003**


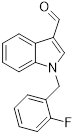

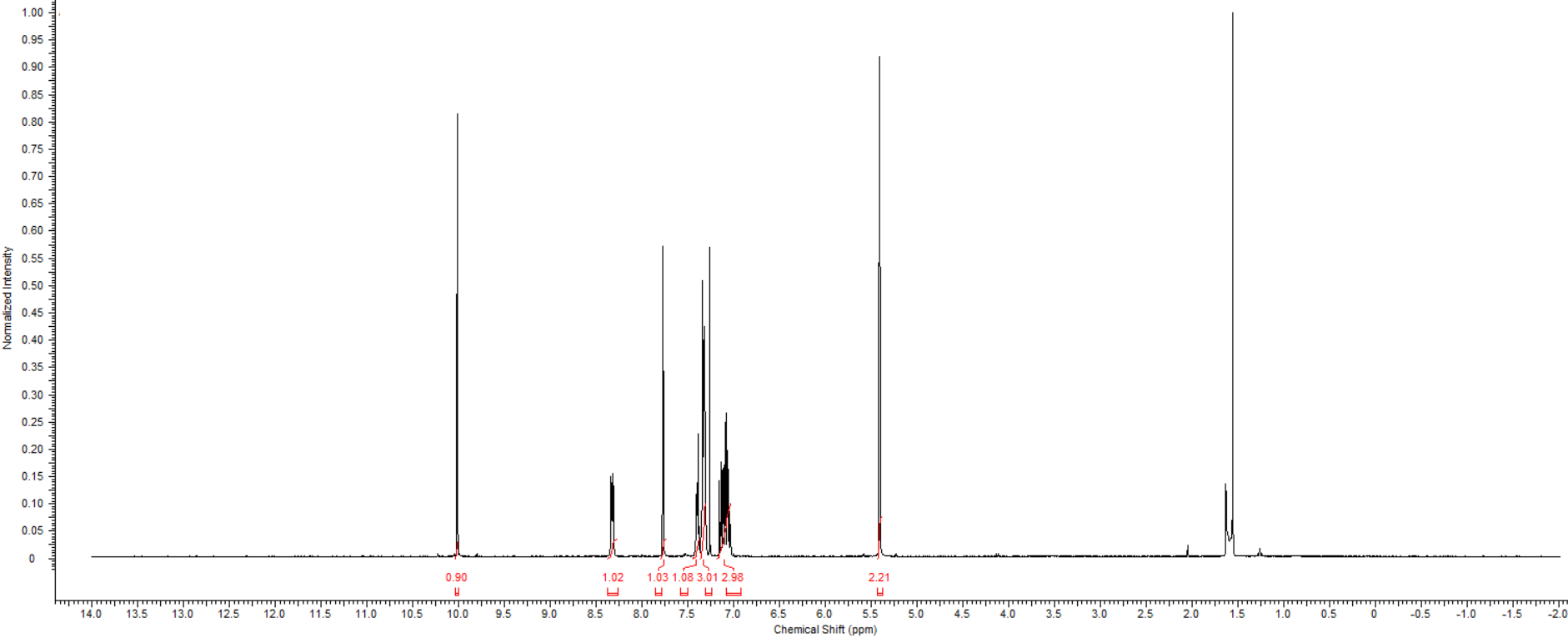


**^13^C NMR of KAM003**


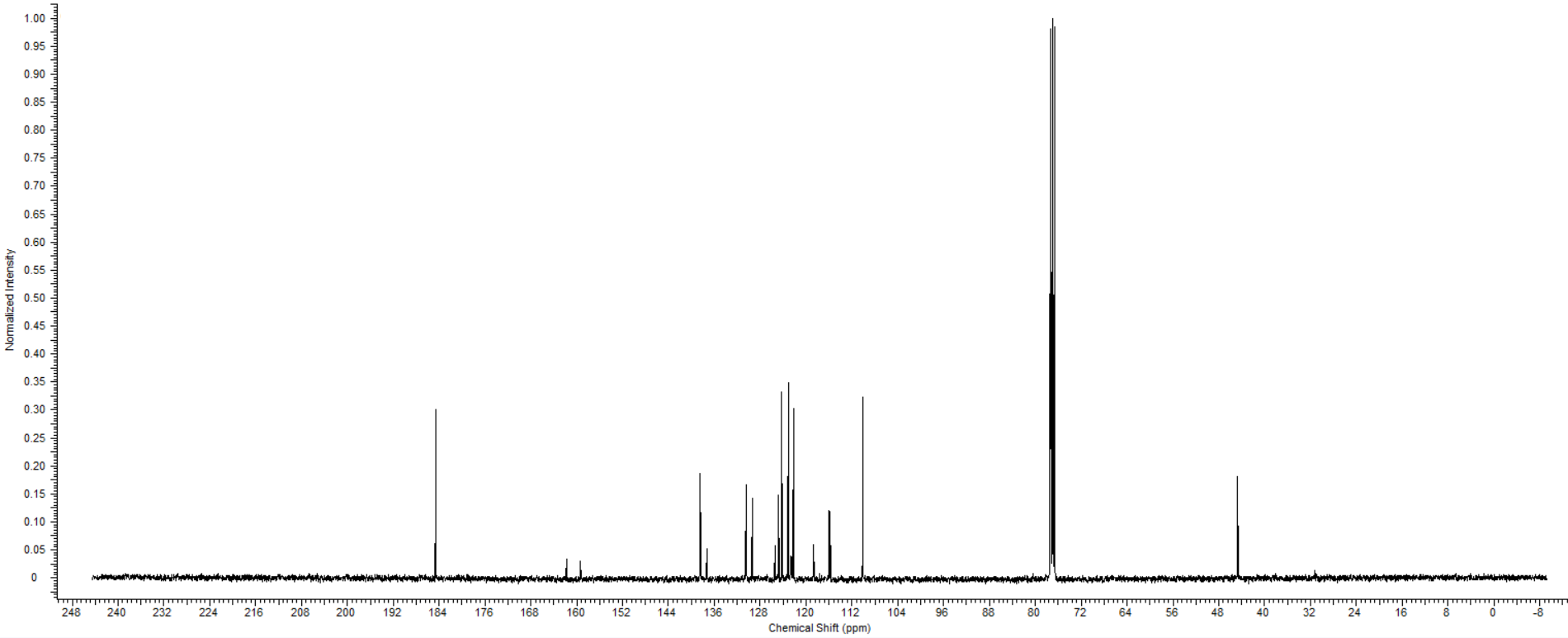

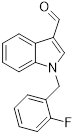


**Structure**

**Identifier – BpinKAM001**1-(4-(4,4,5,5-tetramethyl-1,3,2-dioxaborolan-2-yl)benzyl)-1H-indole-3-carbaldehyde

**Mass Spec**

Chemical Formula: C22H24BNO3

Exact Mass: 361.24186

Molecular Weight: 361.18492

Observed Mass (M + H )

**^1^H NMR** :

(CDCl_3_, 400 MHz) δ 10.00 (s, 1H), 8.33 (d, J = 6.65 Hz, 1H), 7.79 (d, J = 7.83 Hz, 2H), 7.70 (s, 1H), 7.27 - 7.35 (m, 3H), 7.19 (d, J = 7.83 Hz, 2H), 5.37 (s, 2H), 1.33 (s, 12H)

**^13^C NMR:**
(CDCl_3_, 101 MHz) δ 184.6, 138.4, 138.2, 137.4, 135.5, 126.5, 125.5, 124.2, 123.1, 122.2, 110.3, 84.0, 51.1, 30.9, 24.8

Crystal structure:

Link: DOI: 10.5517/ccdc.csd.cc23syc7


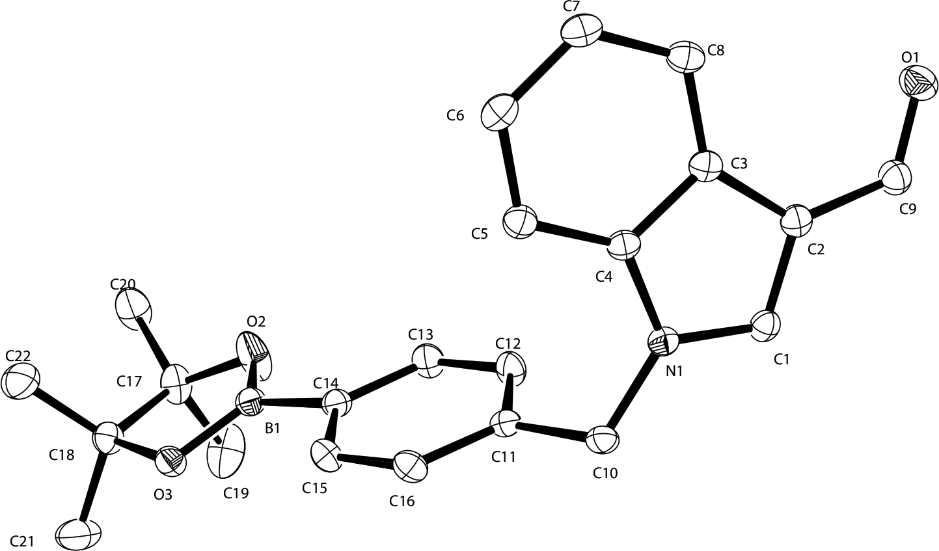


**^1^H NMR of BpinKAM001**


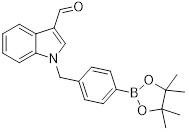

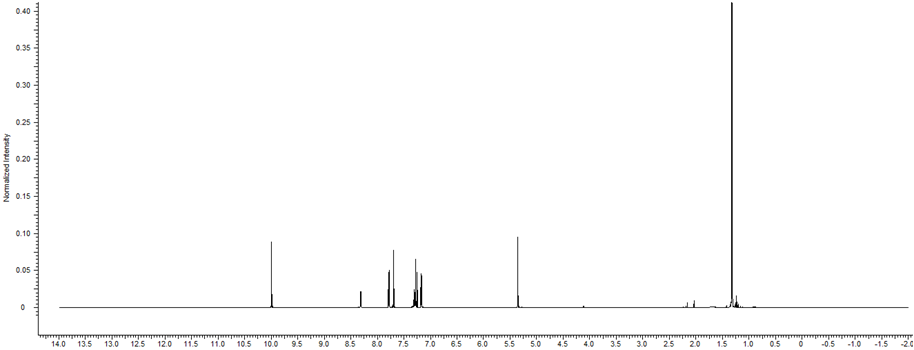


**^13^C NMR of BpinKAM001**


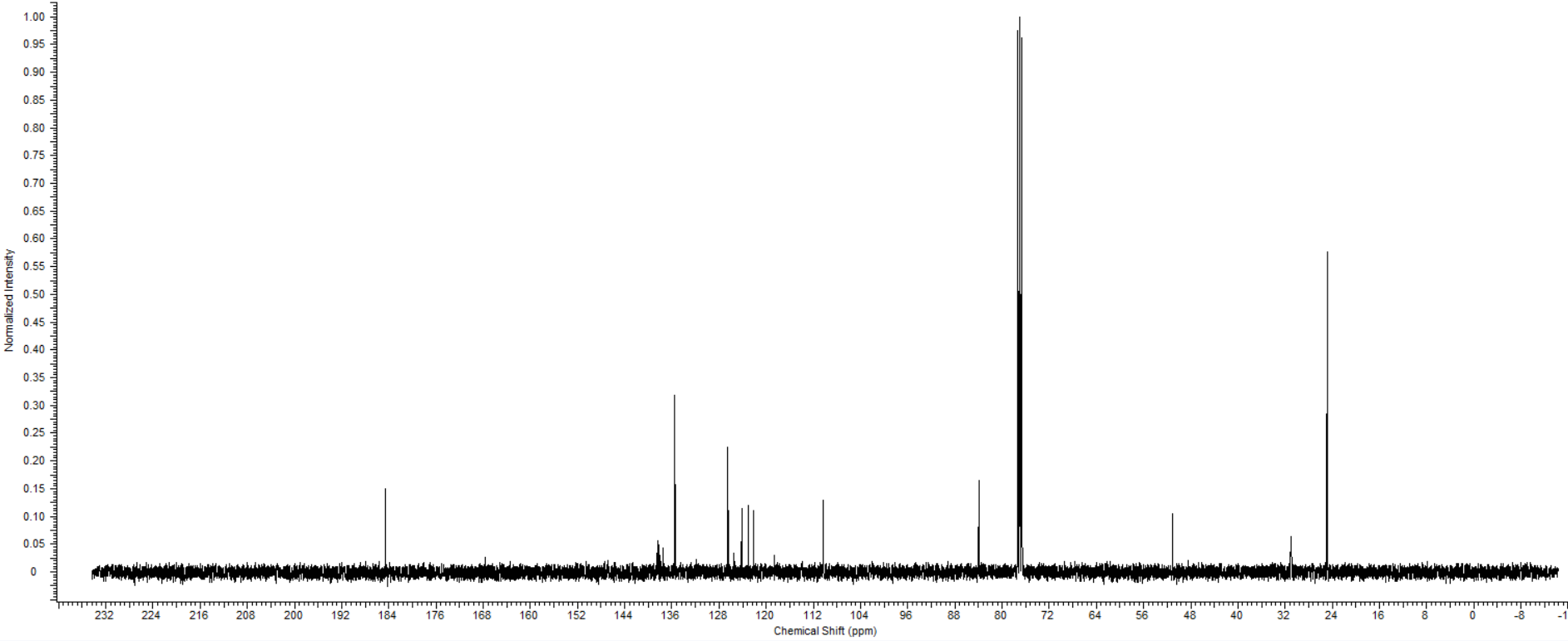

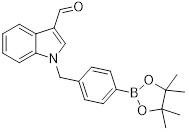


**Structure**

**Identifier – BpinKAM002**1-(3-(4,4,5,5-tetramethyl-1,3,2-dioxaborolan-2-yl)benzyl)-1H-indole-3-carbaldehyde

**Mass Spec**

Chemical Formula: C22H24BNO3

Exact Mass: 361.24186

Molecular Weight: 361.18492

Observed Mass (M + H )

**^1^H NMR** :

(CDCl_3_, 400 MHz) δ 10.00 (s, 1 H), 8.31 (s, 1 H), 7.82 - 7.74 (m, 2 H), 7.41 - 7.27 (m, 4 H), 7.21 (s, 1 H), 5.34 (s, 2 H), 1.35 (s, 12 H)

**^13^C NMR:**
(CDCl_3_, 101 MHz) δ 184.96, 138.42, 137.47, 134.90, 134.46, 133.88, 130.20, 128.59, 125.53, 124.10, 123.04, 122.12, 118.45, 110.36, 84.09, 50.94

Crystal Structure

Link: DOI: 10.5517/ccdc.csd.cc23syhc


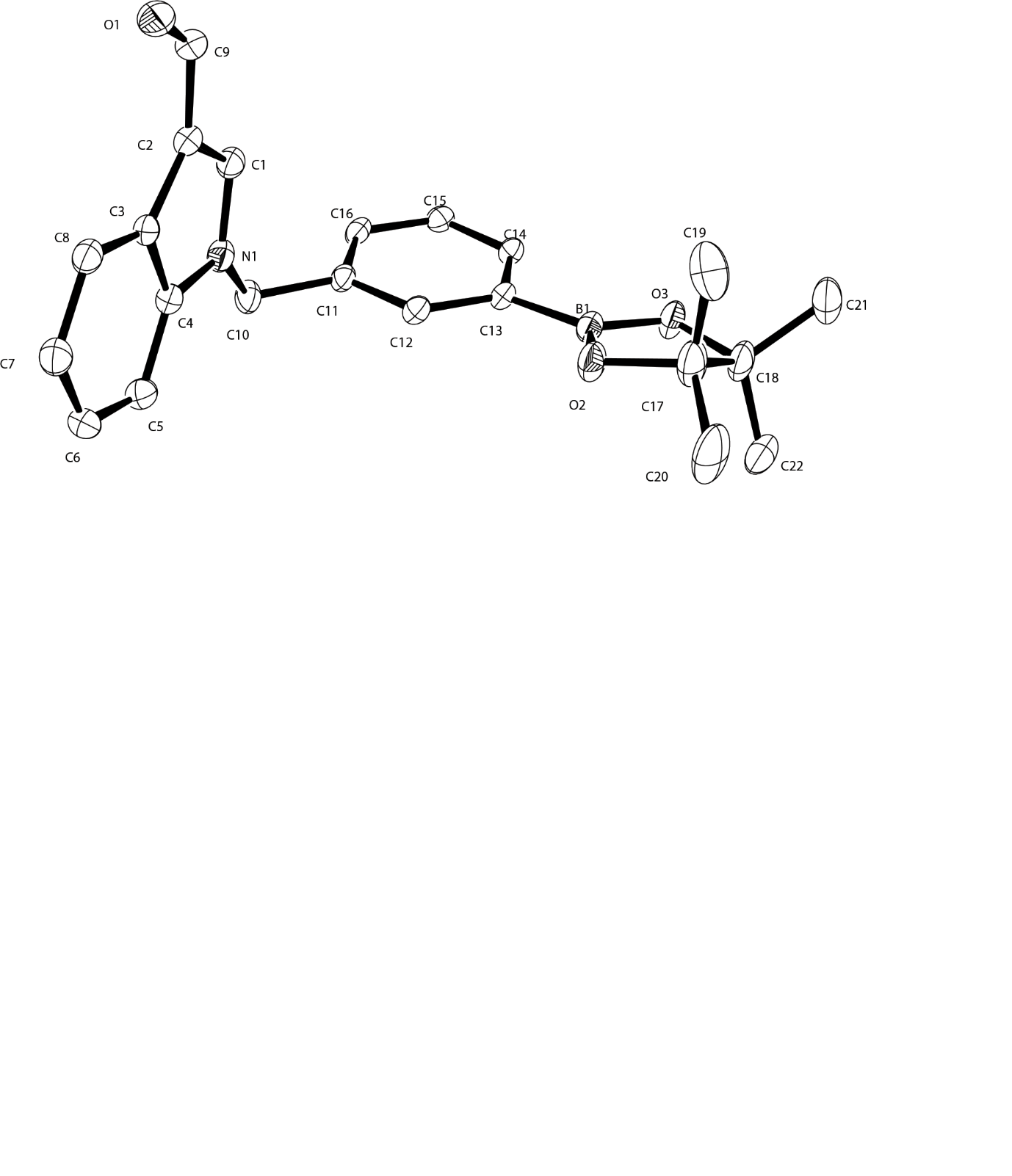


**^1^H NMR of BpinKAM002**


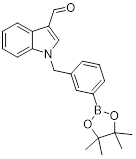

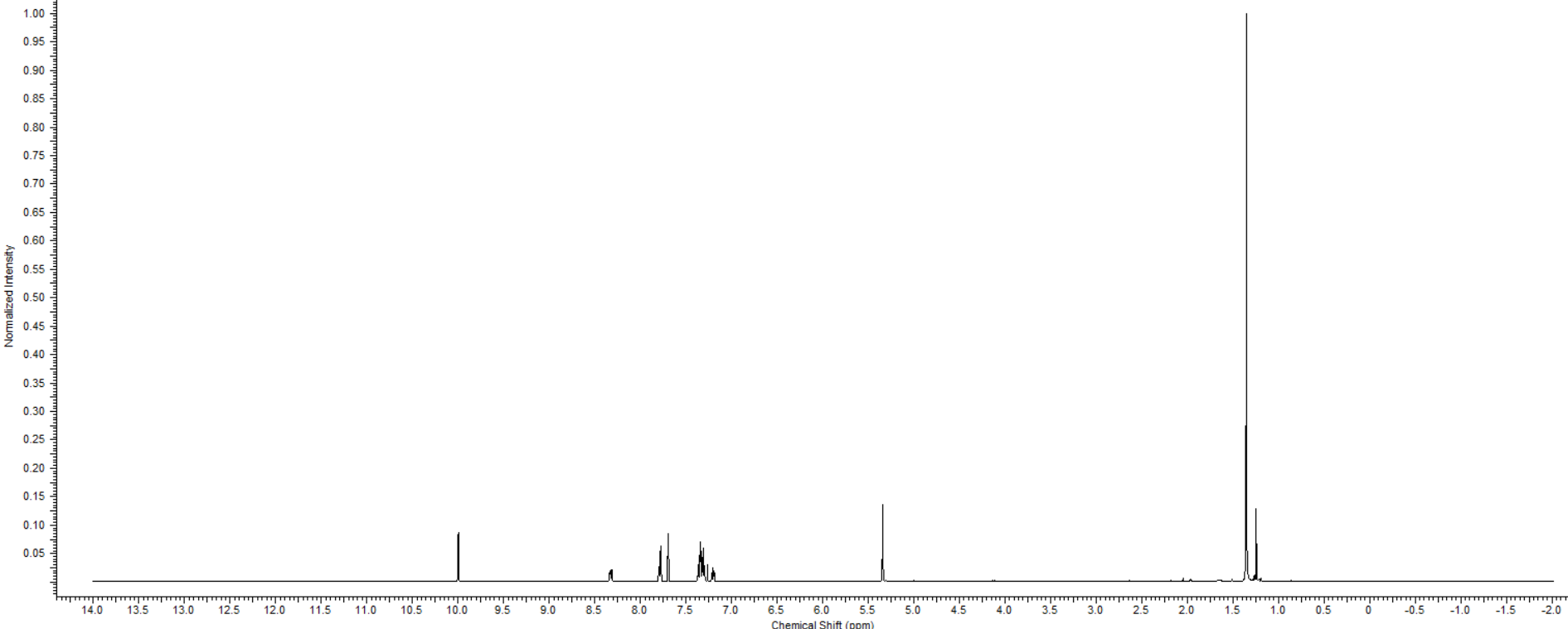


**^13^C NMR of BpinKAM002**


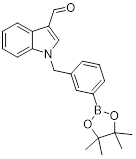

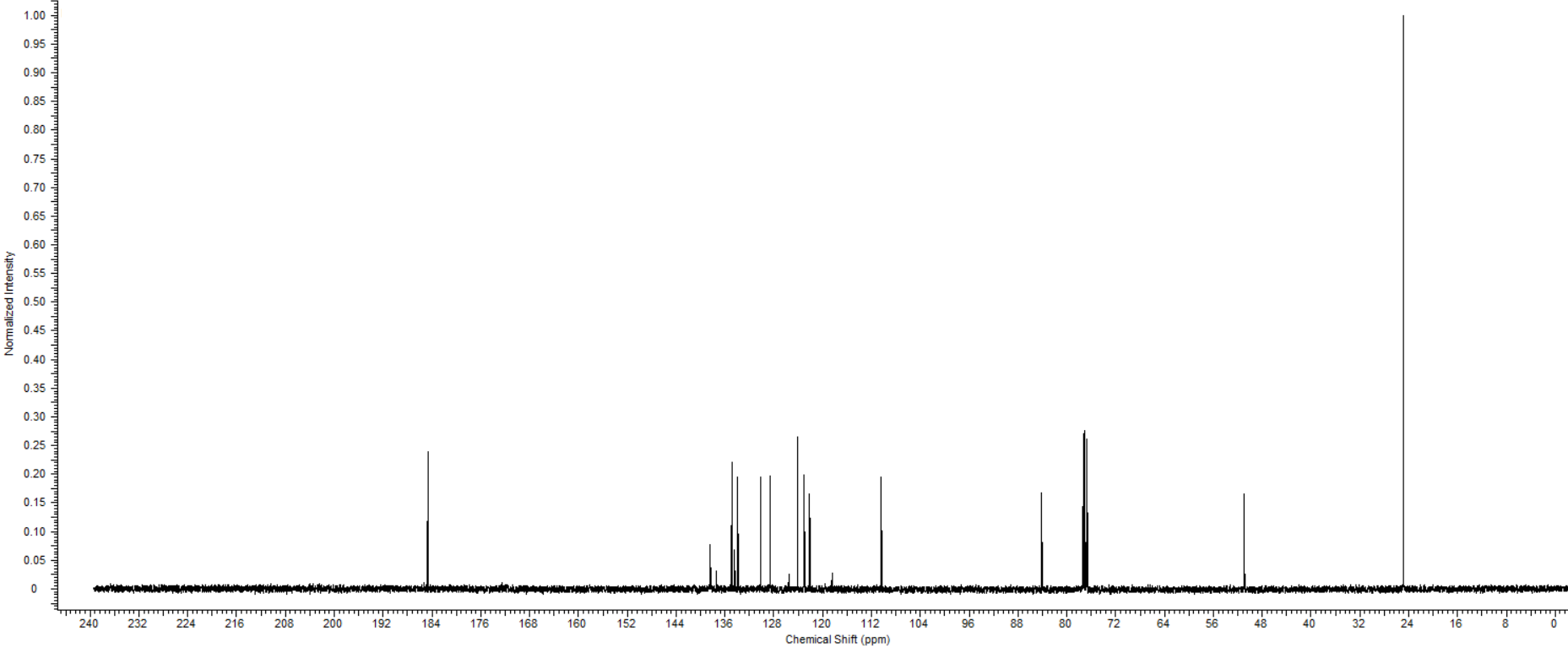


**Structure**

**Identifier – BpinKAM003**1-(2-(4,4,5,5-tetramethyl-1,3,2-dioxaborolan-2-yl)benzyl)-1H-indole-3-carbaldehyde

**Mass Spec**

Chemical Formula: C22H24BNO3

Exact Mass: 361.24186

Molecular Weight: 361.18492

Observed Mass (M + H )

**^1^H NMR** :

(CDCl_3_, 400 MHz) δ 9.94 (s, 2 H), 8.39 - 8.28 (m, 2 H), 7.96 (d, *J* = 6.7 Hz, 2 H), 7.59 (s, 2 H), 7.47 - 7.24 (m, 11 H), 7.01 (d, *J* = 7.0 Hz, 2 H), 1.23 (s, 12 H)

**^13^C NMR:**
(CDCl_3_, 101 MHz) δ 184.6, 141.3, 137.9, 131.8, 128.1, 127.7, 125.4, 123.4, 122.0, 118.1, 110.7, 84.0, 77.3, 77.0, 76.7, 50.3, 24.7

Crystal Structure


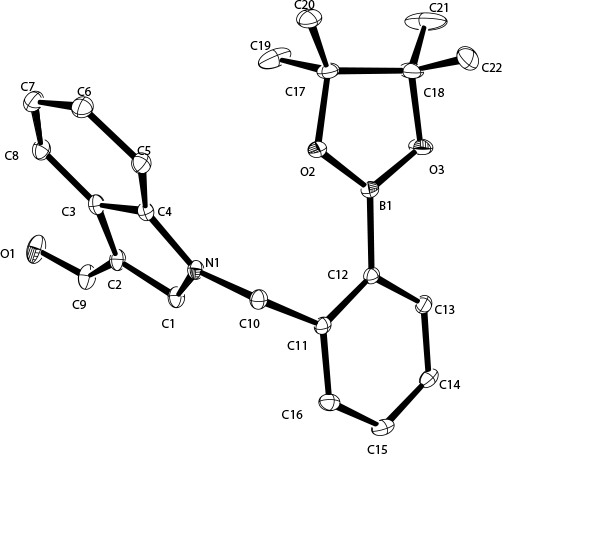
Link: DOI: 10.5517/ccdc.csd.cc23sygb

**^1^H NMR of BpinKAM003**


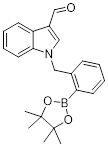

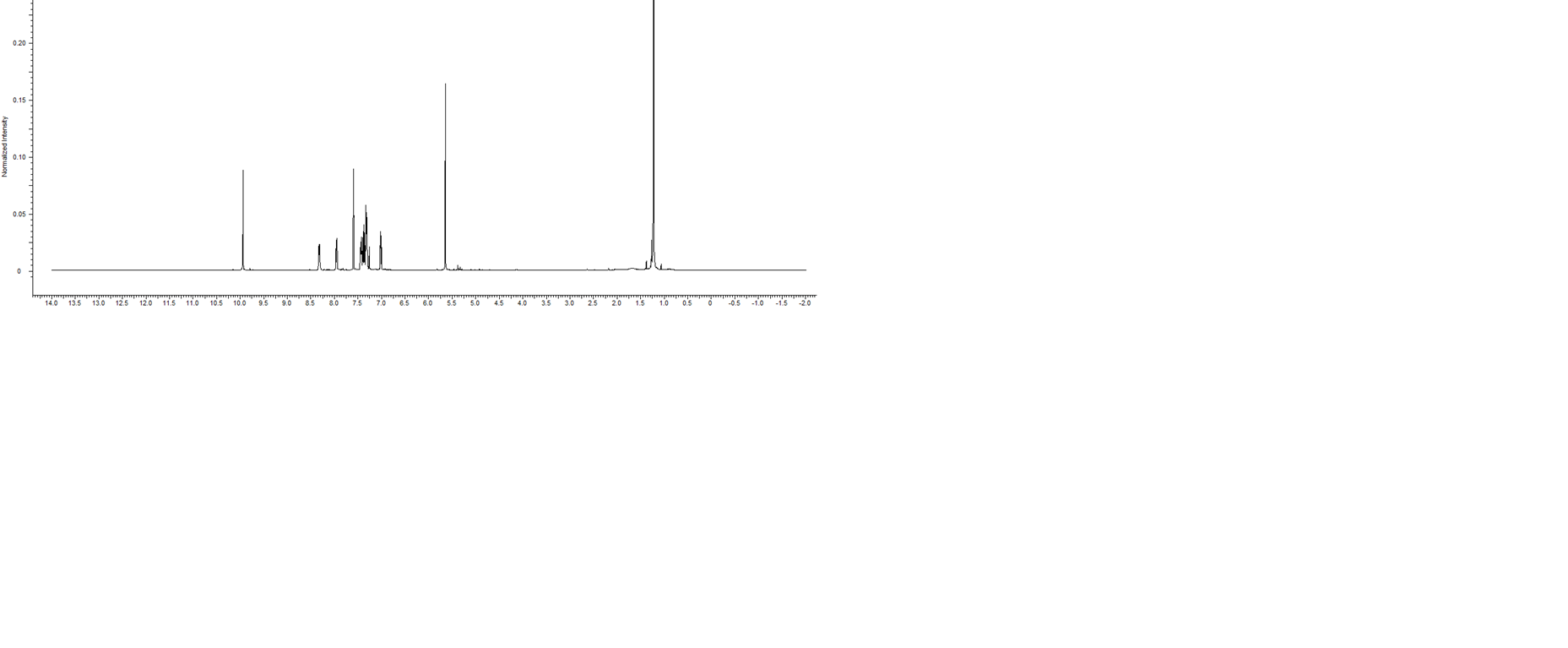


**^13^C NMR of BpinKAM003**


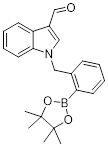

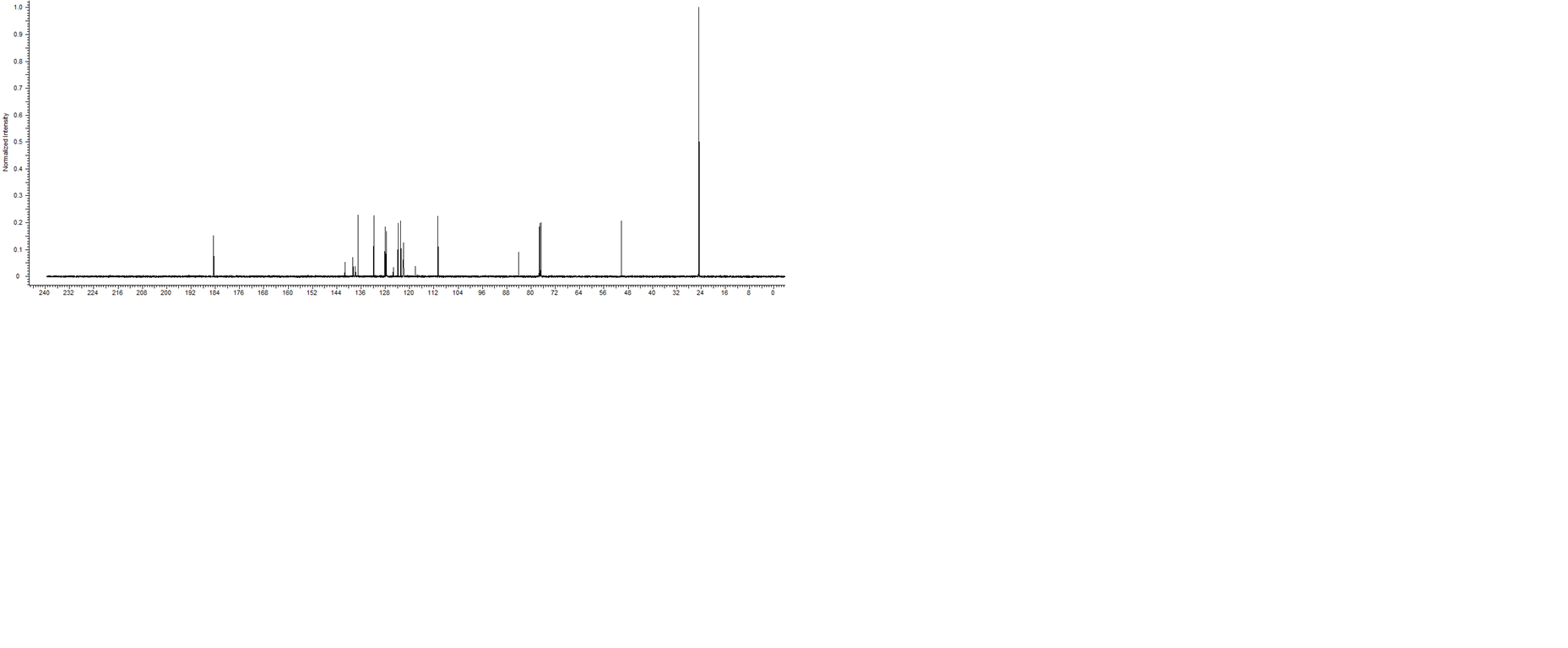


**Characterization of radiolabelled compounds**

*^18^F KAM001*

**Radiolabeling of BpinKAM001**

*Cold Standard Profile:*

Total Ion Count (Mass selected in pink for 254)


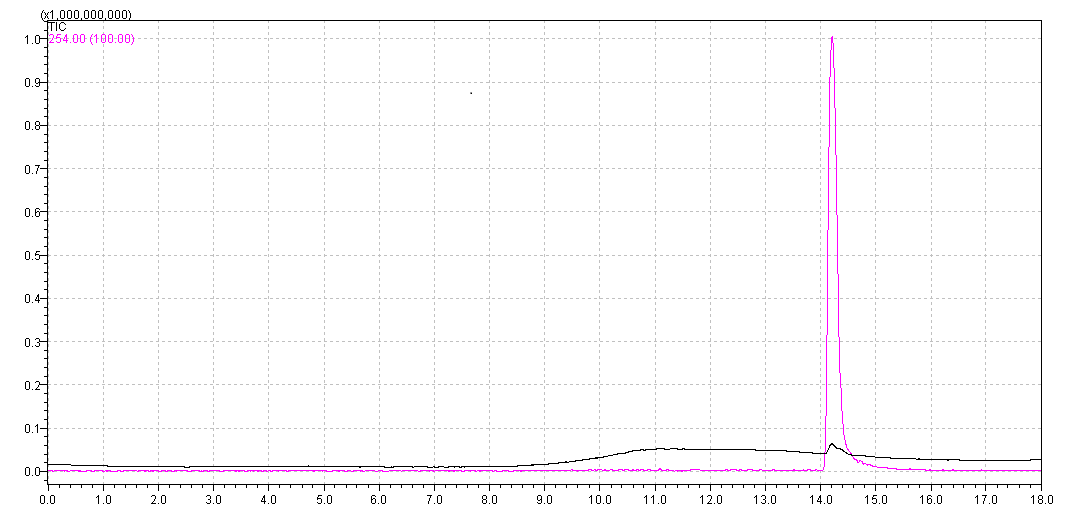


Mass Spectrum


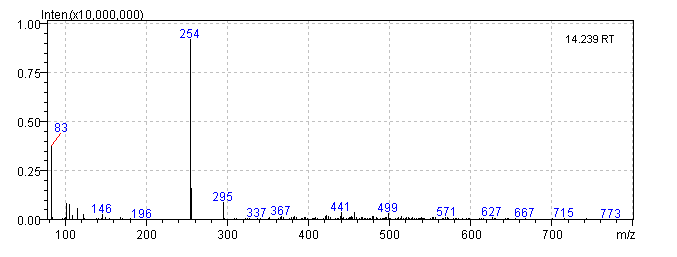

Example of separation radiochromatogram of ^18^F KAM001


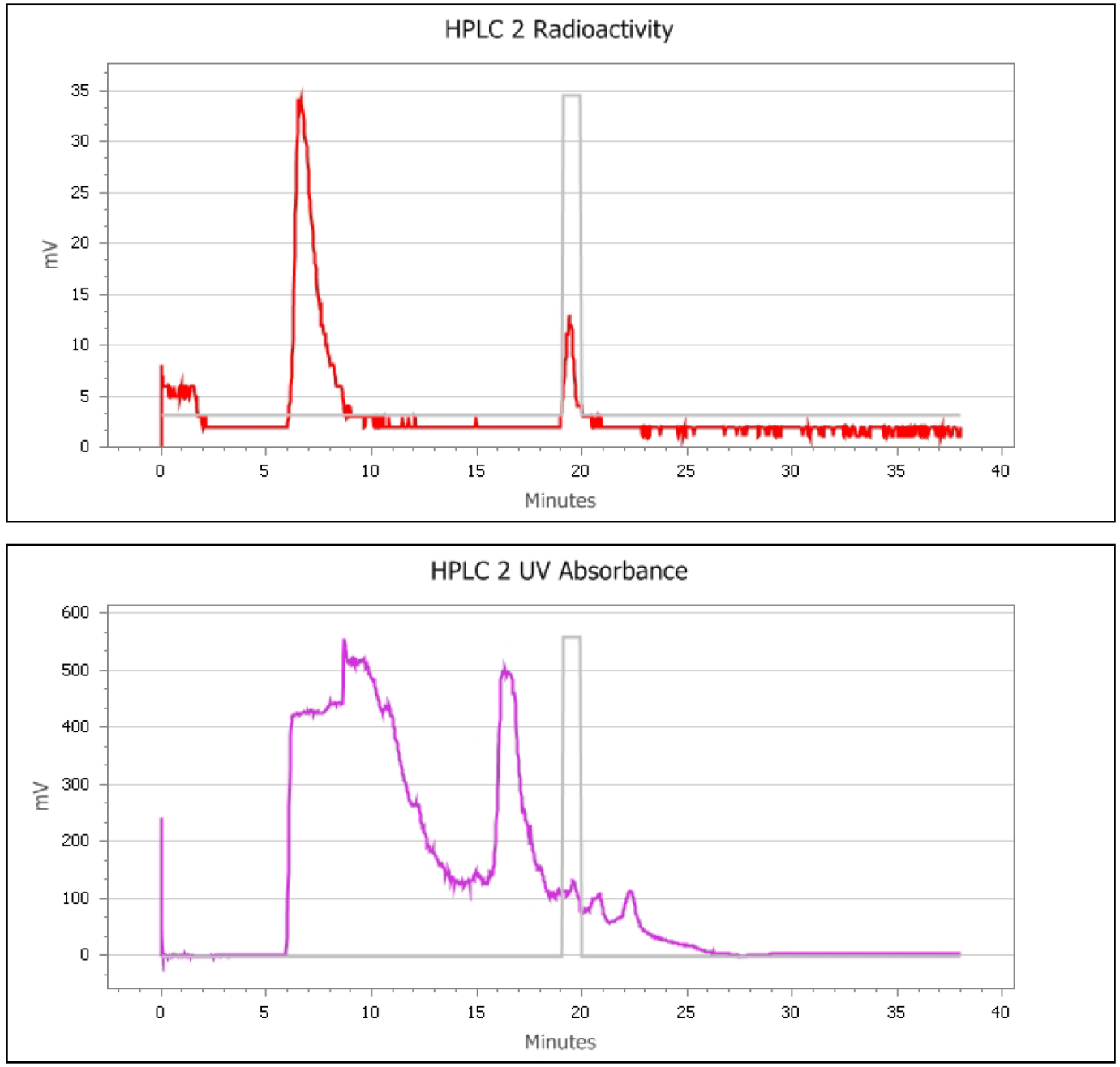


**Radiolabelling of BpinKAM002**

*Cold Standard Profile:*

Total Ion Count (Mass selected in pink for 254)


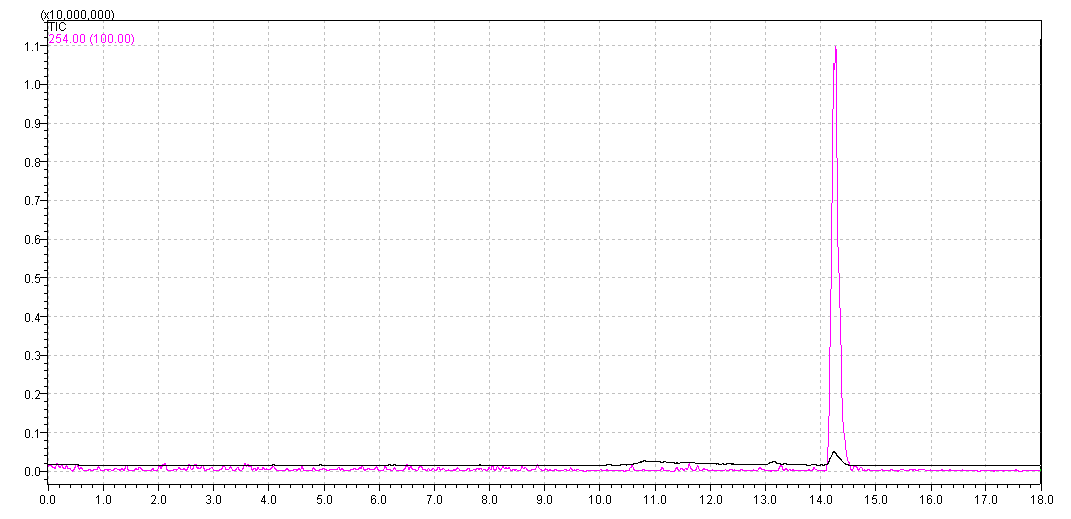


Mass spectrum


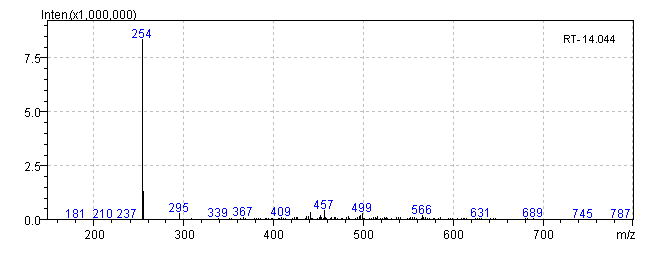

Example of separation radiochromatogram of ^18^F KAM002


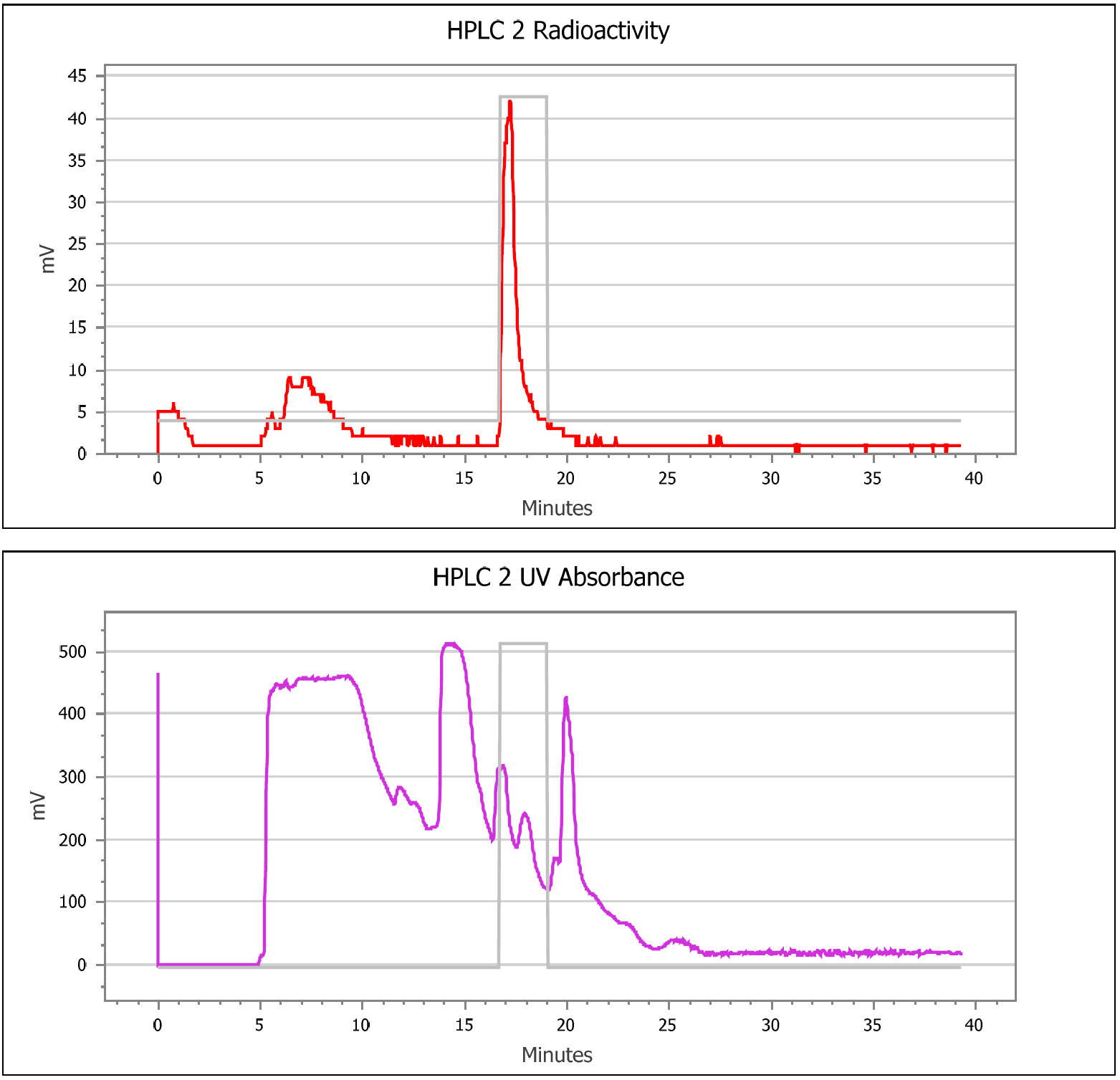


*Molar activity of isolated radioactive products.*

Molar activity was determined by measurement of the UV absorbance of a known amount of radioactivity under identical analytical HPLC conditions used to generate a calibration curve for the corresponding nonradioactive standard.


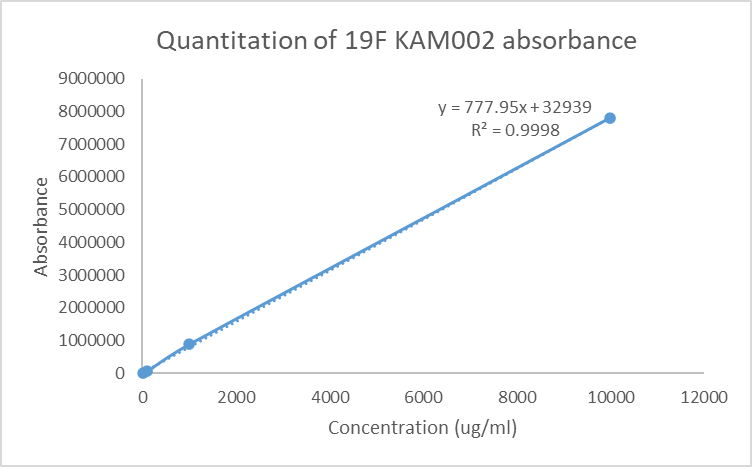


**Radiolabelling of BpinKAM003**

*Cold Standard Prolfile:*

Total Ion Count (Mass selected in pink for 254)


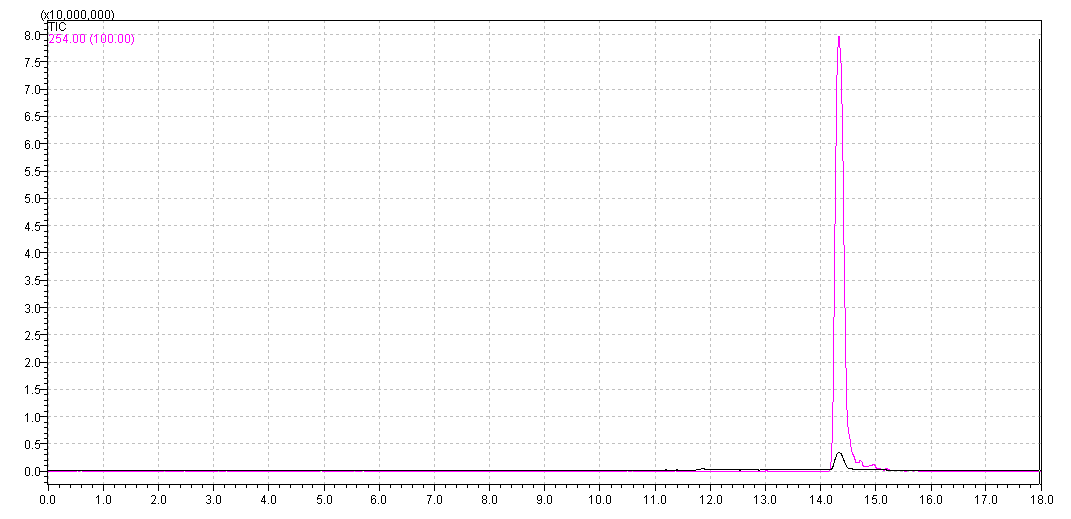


Mass spectrum


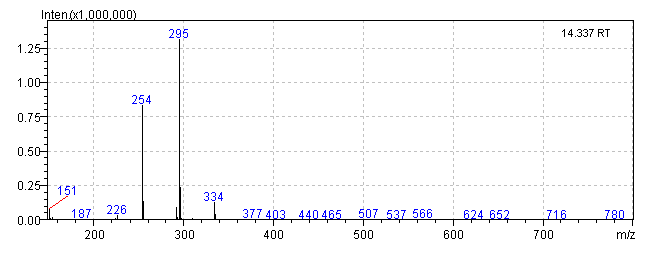

Example of separation radiochromatogram of ^18^F KAM003


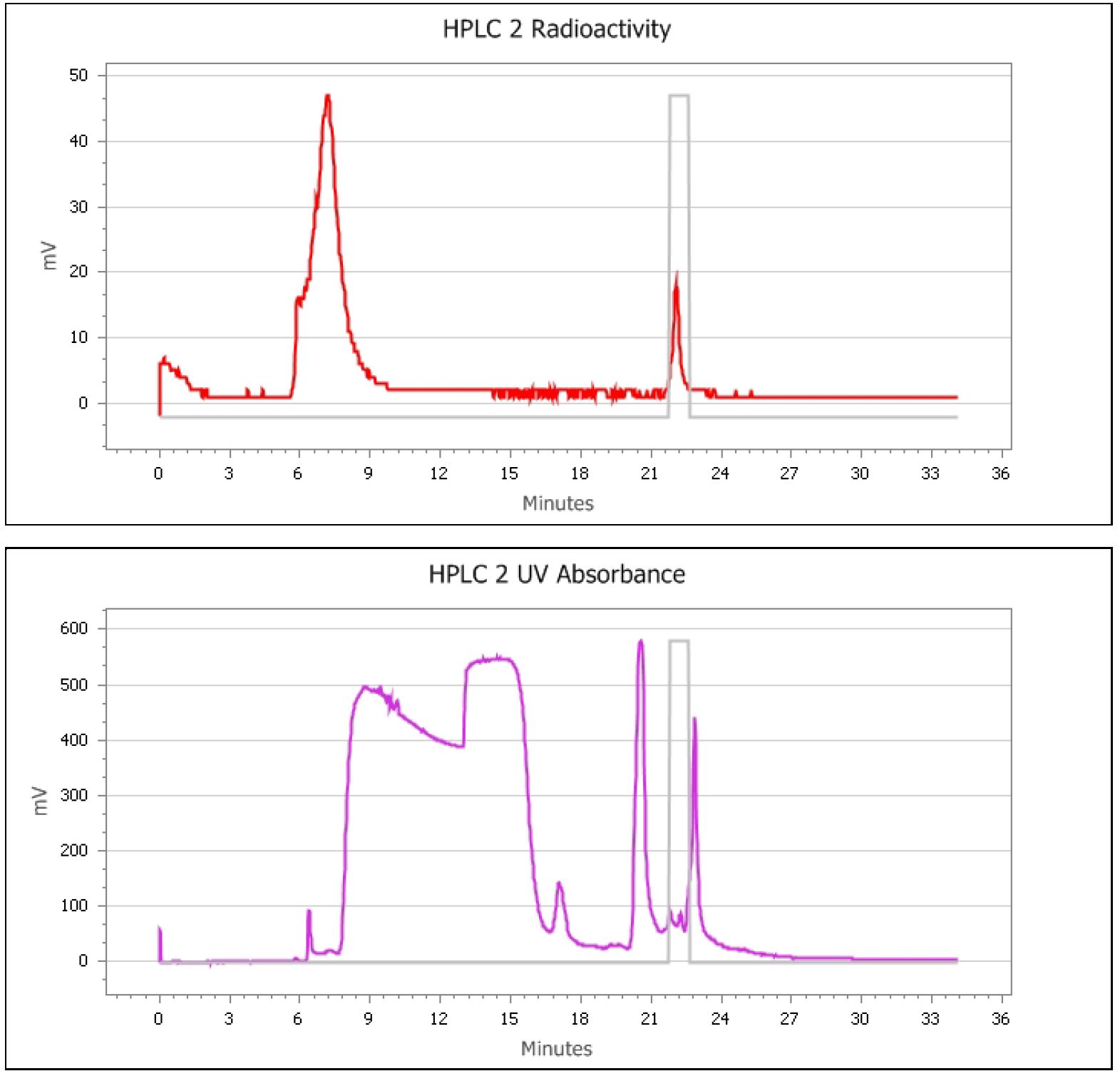


### Potency testing of compounds in MCF-7 and MDA-MB-231 cells

Previous work by Guo and co-workers indicated MCF-7 and MDA-MB-231 cell lines are sensitive and insensitive respectively to Oncrasin like compounds (1). To confirm the cold standards retained the potential for imaging Oncrasin-like activity, toxicitiy was evaluated.

### Maintenance of cell culture

Routine cell culture of the MCF7 cell line was carried out in Dubecco’s modified eagle medium (DMEM) with 10% Bovine Calf Serum and 1% of each of glutamax and pen/strep. In addition to these standard additives, a mixture of 10-2 thioglycerol-α, insulin and hydrocortisone, as described below, was added to the media For experimental work, the same mixture was used with a reduction to 1% from 10% of Bovine Calf Serum.

| Additive | Volume for 10L (ml) |
| --- | --- |
| Thioglycerol-α | 10 |
| Insulin (100U/ml) | 2.5 |
| Hydrocortisone (50mg/ml) | 0.2 |
| Media | 7.3 |

Routine cell culture of the COLO205, MDA-MB-231 cell line was carried out in Roswell Park Memorial Institute medium (RPMI) with 10% Bovine Calf Serum and 1% of each of glutamax and pen/strep. For experimental work, the same mixture was used with a reduction to 1% from 10% of Bovine Calf Serum.

All cells were incubated in tissue culture dishes at 37°C with controlled humidity and 5% CO2 air content.

*Preparation of compounds for treatment*

All research compounds were HPLC purified and then dissolved into DMSO to make 10μM solutions, with the final dilution being performed in the media aliquots used for treatment.

### Sulforhodamine B colorimetric assay

#### Cell Number Titration

Depending on the cell line, between approximately 50,000 and 70,000 cells were plated per well in column 1 of a 96 well plate, with a 1:2 serial dilution performed across columns 1-11. Cells were then prepared in accordance with the SRB assay general procedure outlined below. Cell titration numbers were used for estimating a 70% confluence interval which dictates the number of cells to be plated for drug sensitivity assays.

#### Drug sensitivity assay

Cells were seeded overnight in 100 µL of media with column 12 left as a blank with 1% FCS. The following day 100 µL of drugged-media was used to replace the untreated media. Drug media concentrations were determined by premixed serial dilutions from highest concentration in well 1 to well 10 with a 1:3 dilution across the plate before transferring to cells and performed in triplicate, leaving an “untreated” and a blank. Cells were then prepared in accordance with the SRB assay general procedure outlined below.

#### General procedure

Cells were incubated for 72 hours and then fixed to plates by removal of media and addition of 100 µL of cold 10% trichloroacetic acid (TCA) for 30 – 60 minutes. Cells were washed 3 times with tap water and dried at 37°C before the addition of 50 µl of SRB reagent for 10-15 minutes. SRB reagent was removed and cells were washed in 1% acetic acid 3 times to remove unbound dye before drying at 37°C. Dye was solubilized in 100 µL 10 mM Tris-base with shaking for 5-10 minutes before being read on the plate reader at 540nm, with blank absorbance being subtracted.

Data obtained was processed using Prism software and I50 and LogIC50 values were determined using a 3 point linear regression fit. All IC50 values reported for MCF-7 cell lines and MDA-MB-231 cell lines are absolute IC50 values, showing both 100% survivability and 100% death.


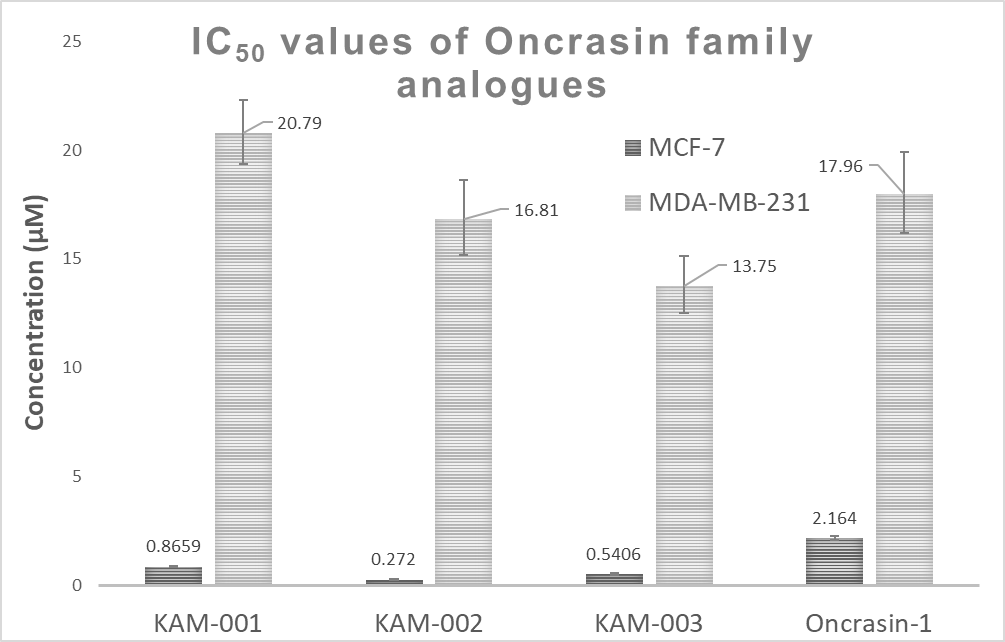

1. Wei X, Guo W, Wu S, Wang L, Lu Y, Xu B, et al. Inhibiting JNK dephosphorylation and induction of apoptosis by novel anticancer agent NSC-741909 in cancer cells. J Biol Chem. 2009;284(25):16948-55.
